# Alterations of the host-lung microbiome metasystem in systemic inflammatory response syndrome is associated with secondary pneumonia

**DOI:** 10.1016/j.xcrm.2026.102919

**Published:** 2026-07-16

**Authors:** Debajyoti Sinha, Melanie Petrier, Florian Pierre Martin, Cecile Poulain, Delphine Flattres Duchaussoy, Chiara Alberti, Stefanie Kreutmair, Jonas Schmid, Susanne Unger, Athanasios Ziogas, Despoina Koulenti, Laia Fernández-Barat, Antoni Torres, Cecile Braudeau, Regis Josien, Burkhard Becher, Mihai G. Netea, Robert P. Dickson, Emmanuel Montassier, Jeremie Poschmann, Antoine Roquilly

**Affiliations:** 1 Nantes Université, Inserm, CHU Nantes, Center for Research in Transplantation and Translational Immunology, UMR 1064, 44000 Nantes, France; 2 Nantes Université, CHU Nantes, INSERM, Service d’Anesthesie Réanimation, CIC 1413, Immuno-infectiologie, 44000 Nantes, France; 3 Institute of Experimental Immunology, University of Zurich, 8057 Zurich, Switzerland; 4 Department of Medical Oncology and Hematology, University Hospital Zürich, Rämistrasse 100, 8091 Zürich, Switzerland; 5 The LOOP Zurich, University of Zurich, Zurich, Switzerland; 6 Department of Internal Medicine and Radboud Center for Infectious Diseases, Radboud University Medical Center, Nijmegen, the Netherlands; 7 Critical Care Department, King’s College Hospital NHS Foundation Trust, London, UK; 8 UQ Centre for Critical Research (UQCCR), The University of Queensland, Brisbane, QLD, Australia; 9 Medical School, University of Thessaly, Larissa, Greece; 10 Faculty of Health Sciences, University of Ioannina, Ioannina, Greece; 11 Research Foundation Clinic Barcelona (FRCB)- August Pi i Sunyer Biomedical Research Institute (IDIBAPS), Centro de Investigación Biomédica en Red de Enfermedades Respiratorias (CIBERES), Barcelona, Spain; 12 Department of Pulmonology, Thorax Institute, Hospital Clinic de Barcelona, Barcelona, Spain; 13 Department of Medicine, Faculty of Medicine and Department of Microbiology, Faculty of Pharmacy & Food Sciences, University of Barcelona, Barcelona, Spain; 14 FERS ATS Fellow University of Barcelona, School of Medicine, CIBERES.IDIBAPS, Barcelona, Spain; 15 Nantes Université, CHU Nantes, Laboratoire d’Immunologie Biologique, CIMNA, 44000 Nantes, France; 16 Department of Microbiology and Immunology, University of Michigan Medical School, Ann Arbor, MI, USA; 17 Weil Institute for Critical Care Research and Innovation, Ann Arbor, MI, USA; 18 Division of Pulmonary and Critical Care Medicine, Department of Internal Medicine, University of Michigan Health System, Ann Arbor, MI, USA; 19 Nantes Université, CHU Nantes, Service des Urgences, 44000 Nantes, France; 20 Department of Microbiology and Immunology, The University of Melbourne, The Peter Doherty Institute for Infection and Immunity, Melbourne, VIC 3000, Australia

**Keywords:** respiratory microbiome, metabolome, immune therapy, pneumonia, SIRS, precision medicine, sepsis, interferon

## Abstract

Host-respiratory microbiome interplay is vital to lung homeostasis. Systemic inflammatory response syndrome (SIRS) is an intense alteration in host status that necessitates rapid microbiome adaptation to avoid respiratory complications. Using longitudinal multi-omic data from patients with SIRS, we confirm that the respiratory microbiome, blood metabolome, and immune cells form a dynamic metasystem and define a metacluster with distinct T/B cell trafficking, anaerobic bacteria, high tyrosine metabolism, and low fatty acid biosynthesis. This metacluster status can serve to classify the severity of alterations in host-lung microbiome interactions as moderate or severe and to predict pneumonia and mortality. We demonstrate the robustness of these findings in an independent, randomized controlled trial and propose that interferon-γ treatment may benefit patients with severe metacluster alterations but harm those with moderate alterations. Our study supports the concept of the host-respiratory microbiome as a dynamic metasystem, in which specific alterations are associated with pneumonia and responses to interferon-γ treatment.

## Highlights

• Respiratory microbiome, metabolome, and immune cells form a dynamic metacluster
• Lymphocyte trafficking, anaerobic bacteria, and fatty acid biosynthesis correlate
• 4-Factor score can classify the severity of these metacluster alterations
• 4-Factor score can identify high-risk patients and responders to immunotherapy

## Graphical abstract

**Figure:**
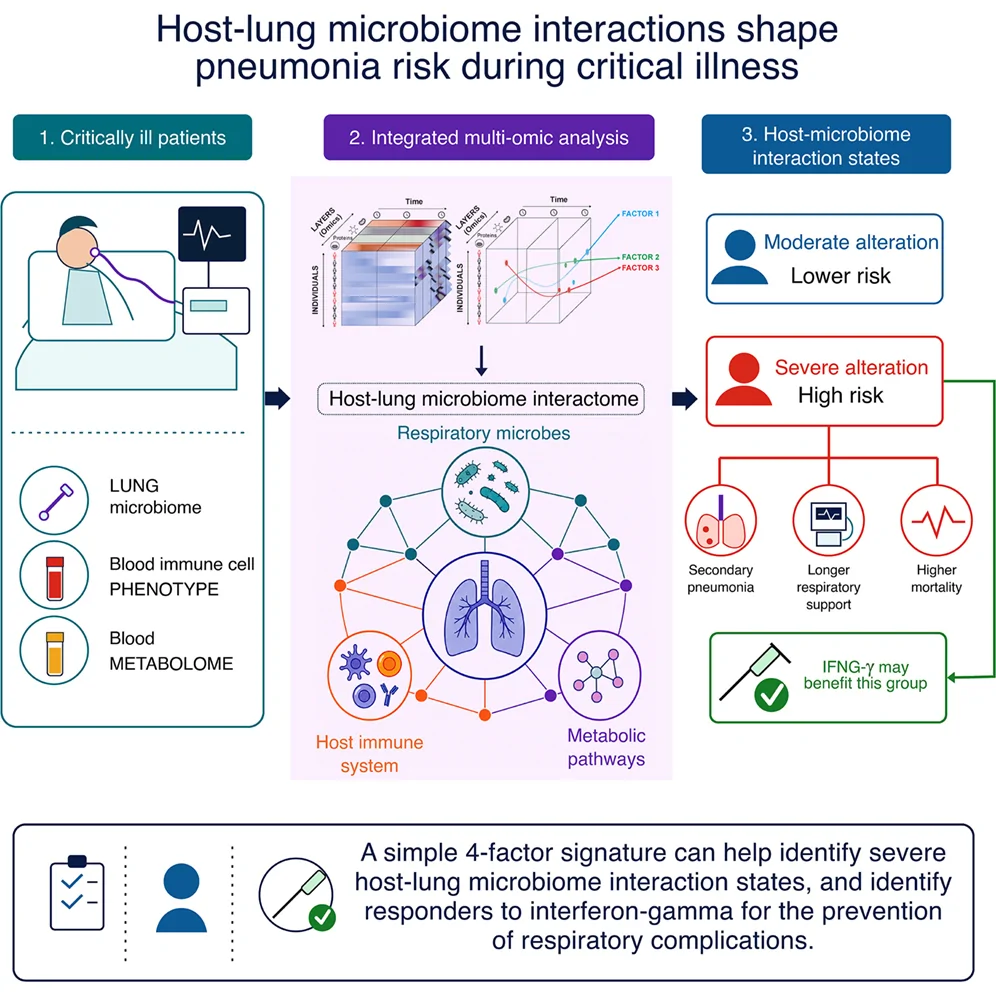

## Teaser

Roquilly et al. identify a metacluster involving lymphocyte trafficking, lung anaerobic bacteria, tyrosine metabolism, and fatty acid biosynthesis, which is involved in lung homeostasis and susceptibility to pneumonia. This metacluster can be used as a classification tool to assess individual respiratory risk and predict response to interferon-γ treatment.

## Introduction

Despite the advances in prevention and treatment, up to 30% of critically ill patients develop hospital-acquired pneumonia (HAP),^1^^,^^2^^,^^3^ and a complicated trajectory toward acute respiratory failure (acute respiratory distress syndrome [ARDS]) is found in up to 30% of critically ill patients in intensive care units (ICUs).^3^^,^^4^^,^^5^^,^^6^ Increasing our understanding of the mechanisms underlying respiratory complications in critically ill patients should enable the development of personalized, innovative therapeutics for HAP and ARDS.

Commensal respiratory microbes are crucial for maintaining lung homeostasis.^7^^,^^8^ The intricate interplay between the host and the respiratory microbiome must be finely tuned to avoid respiratory complications. In particular, perturbations of the lung environment, whether from endogenous events such as systemic inflammatory response syndrome (SIRS) or exogenous factors such as microaspirations of gastric contents or antimicrobial therapy, necessitate rapid bacterial adaptation. The impact of SIRS on gut commensal microbes has been the subject of extensive research.^9^^,^^10^ Even though pneumonia is the most frequent complication in patients with SIRS,^11^ data on transkingdom communication between the host and respiratory microbiome are scarce.^12^^,^^13^^,^^14^

We performed a longitudinal high-throughput characterization of the host-lung microbiome metasystem in a prospective cohort of critically ill patients with SIRS to discover host-respiratory microbiome metaclusters and test their associations with HAP, duration of respiratory failure, and death. Then, we developed an easy-to-use score for individual classification of the severity of alterations in host-lung microbiome interactions and tested the utility of this classification for predicting outcomes and treatment response in an independent multi-center cohort.

## Results

### Study design

**Figure 1:**
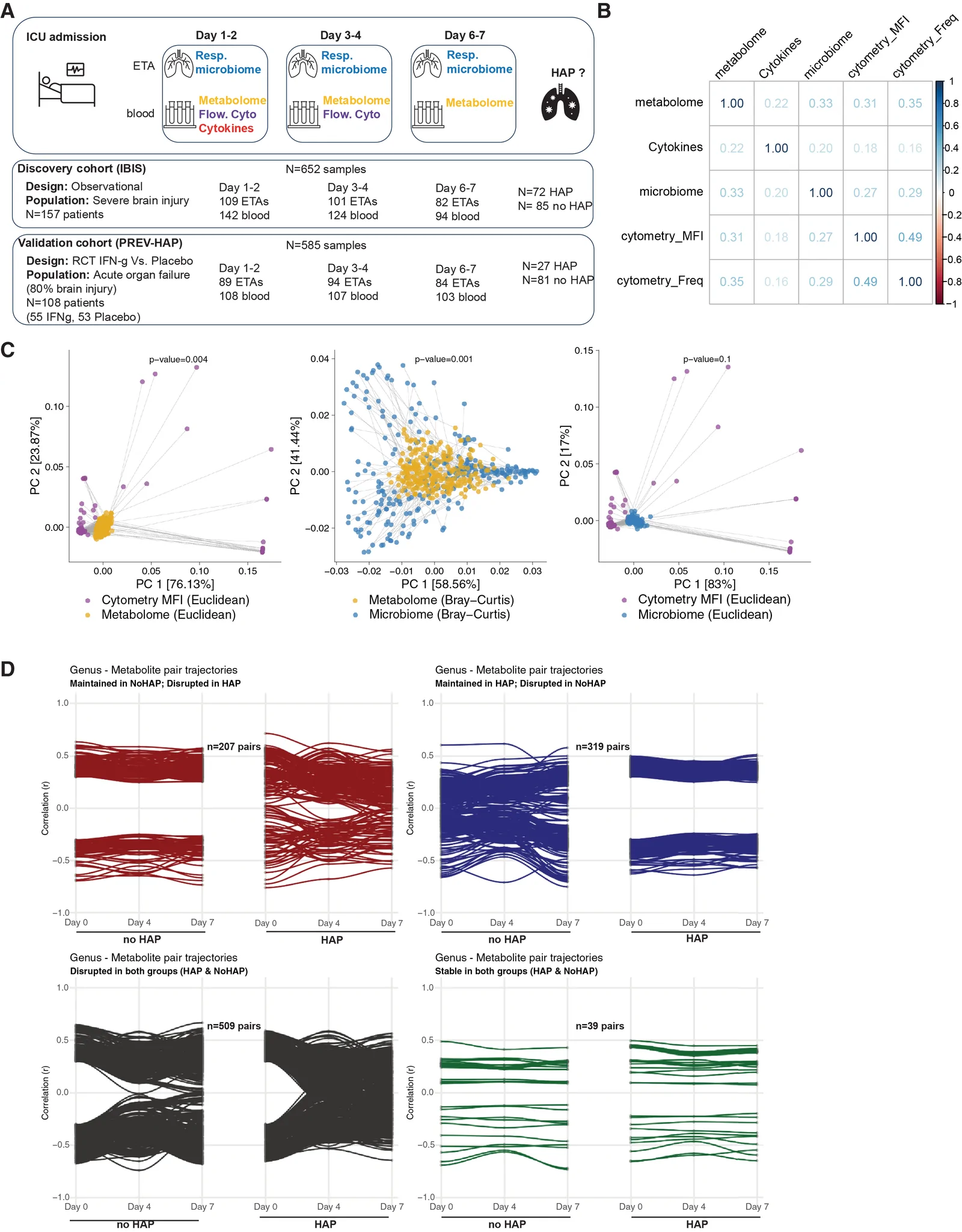
Alterations of the host-lung microbiome metasystem are associated with HAP (A) Study design showing the exact number of patients and samples stratified by time. (B) Correlation table of omic layer compositions. (C) Procrustes analyses of the superimpositions of principal-component analyses from cytometry (MFI), metabolome, and respiratory microbiome. (D) Time courses of microbiome and metabolome correlation values in patients with or without HAP.

Clinical and biological data used in this study were obtained from two datasets (Figure 1A): (1) the Immunology and Brain Injury Study (IBIS) study, an observational cohort to study the pathophysiology of HAP in patients with severe brain injury (Glasgow coma scale <12 and requiring invasive mechanical ventilation) hospitalized in two ICUs in France, served as the discovery cohort^15^^,^^16^ and (2) the PREV-HAP study, a phase 2 randomized, double-blind study comparing interferon-gamma 1b and placebo for the prevention of HAP in critically ill patients (one or more acute organ failure and requiring invasive mechanical ventilation) performed in 9 French centers,^17^ served as the validation cohort. In both studies, patients were clinically followed up for 90 days for respiratory complications (HAP, ARDS, and mechanical ventilation support) and survival, and blood (plasma and peripheral blood mononuclear cells [PBMCs]) and respiratory samples (endotracheal aspirates [ETAs]) were longitudinally collected (upon ICU admission and then days 3–4 and 6–7). Based on sample availability, we selected 157 patients with brain injury from the IBIS for the discovery cohort and 108 patients from the PREV-HAP study. The baseline characteristics and clinical outcomes are described in Table S1. The frequency of HAP was higher in the IBIS cohort than in the PREV-HAP study (46% vs. 25%, respectively). In this study, respiratory complications include HAP, as well as non-infectious respiratory outcomes such as ARDS and prolonged mechanical ventilation. Importantly, all ARDS cases occurred in patients who were HAP positive, so ARDS was not analyzed as a separate non-HAP respiratory complication in this dataset.

Because antibiotic exposure is a major determinant of respiratory microbiome composition in ICU patients, we summarized the antibiotic exposure at each sampling time point in both cohorts, stratified by HAP, ARDS, and death, and visualized patient-level exposure timelines (Figures S1 and S2). Pre-admission antibiotic exposure was not available in a standardized manner and was thus analyzed. Antibiotic exposure increased over time and was more frequent at later time points in patients who developed HAP or ARDS, whereas associations with death were less consistent.

### Sampling protocols and experimental approaches

ETAs were frozen at −80°C for later microbiome analysis by 16S RNA sequencing (Figure S3, 103 genera, 3 visits). Blood samples were processed for blood cell counts, with plasma and PBMCs isolated and cryopreserved for later analysis by flow-injection mass spectroscopy for metabolomics (516 variables, 3 visits, Figure S4), Luminex for inflammatory cytokine blood concentration measurements (22 variables, 1 visit on day 0, Figure S5), and high-parametric single-cell spectral flow cytometry (2 visits on days 0 and 3–4, Figures S6 and S7; Tables S2, S3, and S4). This immune profiling approach enabled us to assess (1) the overall lymphocyte and myeloid composition of PBMCs (Cytometry_freq; 22 variables) and the expression of functional markers among a range of immune populations (Cytometry_MFI, 264 variables). For metabolomics, putative annotations were generated based on compounds in the Human Metabolome Database, KEGG, and ChEBI databases using accurate mass per charge and isotopic correlation patterns (Table S5 for human-readable annotation). All samples were quality screened, resulting in the inclusion of 652 samples from the IBIS cohort and 585 from the PREV-HAP study.

### SIRS-associated disruption of the host-lung microbiome metasystem differs by HAP status

While the gut microbiota is interconnected with the systemic immune response in critically ill patients,^10^ we questioned whether this is also true for the lung microbiota, which has much lower biomass. First, we assessed the concordance between the omic layers of the respiratory microbiome (ETAs) and peripheral blood metabolome, cytokine, and cytometry compositions by correlating sample similarity structures derived independently for each dataset. For each omic, a sample-by-sample distance matrix was computed using inter-omic kernel integration to quantify cross-omic concordance (see STAR Methods, Figure 1B). Moreover, we performed Procrustes analyses to assess whether the principal-component structures of different omic layers were aligned across samples, reflecting coordinated interpatient variation between compartments. Procrustes analysis has been developed to assess the congruence of two-dimensional shapes obtained by superimposing principal-component analyses from two different datasets. We found significant alignment between the blood metabolome and both blood cytometry and respiratory microbiome principal-component structures (Figure 1C), indicating that variation in circulating metabolites tracks with variation in immune phenotypes and respiratory microbiome composition across patients. This alignment does not imply direct causality but supports the interpretation that these host and microbial compartments do not vary independently during critical illness, consistent with the concept of an interconnected metasystem. Finally, we identified and studied the time course of 1,074 pairs of bacterial genus and blood metabolite with significant correlations (Figure 1D). We found that 1,035 (96.4%) microbiome-metabolite pairs changed over time, indicating that host lung microbiome interactions are dynamically remodeled during critical illness in patients with SIRS. Within this shared inflammatory context, distinct temporal patterns of genus-metabolite relationships were observed in patients who subsequently developed HAP compared with those who did not. Interestingly, 509 pairs (47.4%) were altered in both patients with and without HAP, while 207 pairs (19.3%) were altered specifically in patients with HAP and 319 pairs (29.7%) were altered only in patients without HAP (Figure 1D). Our findings reveal that SIRS disrupts the host-respiratory microbiome metasystem and suggest that its alterations differ between patients who will develop HAP and those who will not.

### Definition of multi-omics factors associated with respiratory complications in critically ill patients

**Figure 2:**
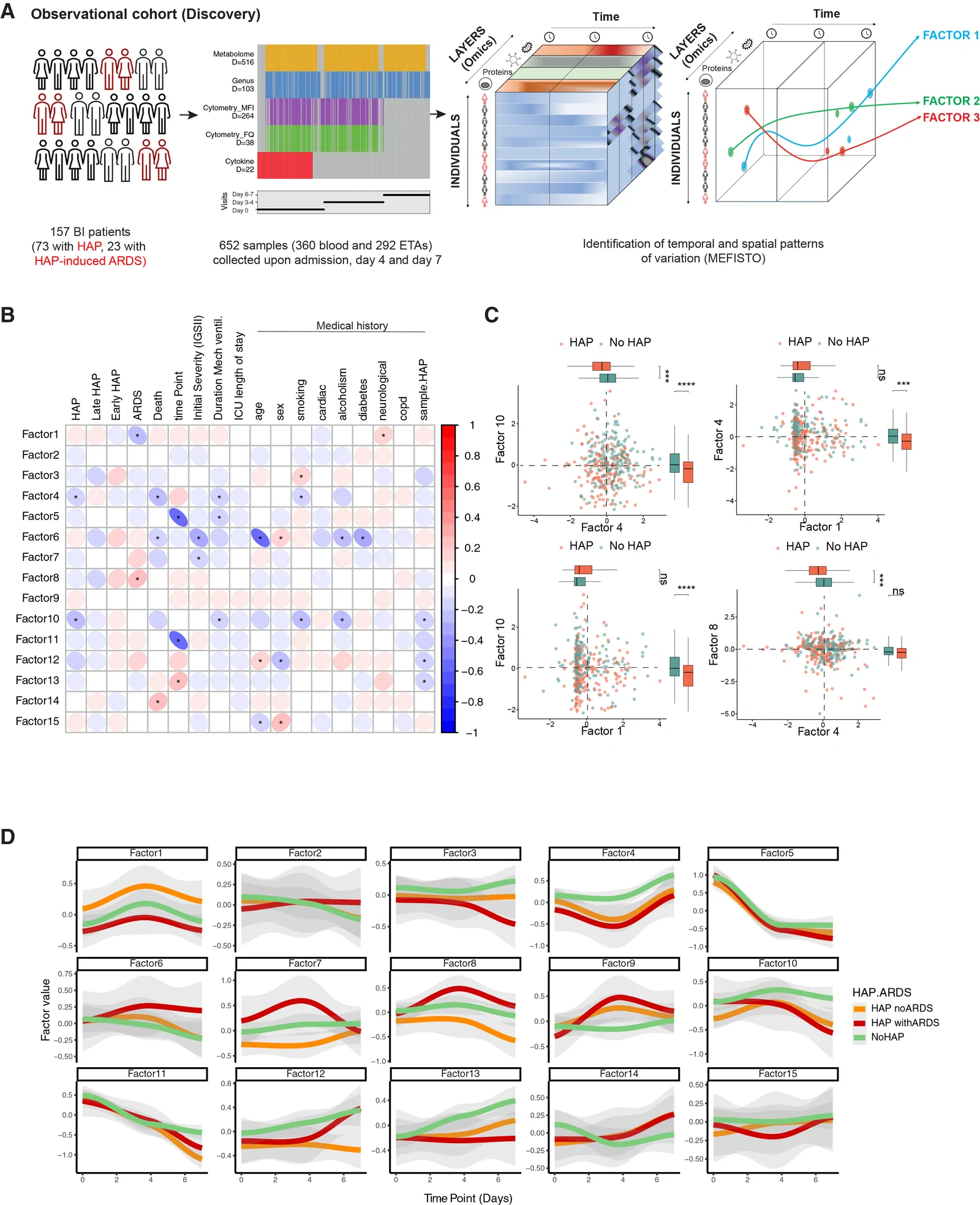
MEFISTO factor associations with HAP group (A) Identification of temporal multi-layer patterns of variation by MEFISTO, and association with clinical outcomes in 157 brain-injured patients with longitudinal endotracheal blood and ETA sampling for respiratory microbiome, circulating metabolome, inflammatory cytokines, and immune phenotyping analysis. (B) Top 15 correlations of MEFISTO factors with clinical traits. HAP samples are significantly associated with Factors 4 and 10. (C) Pairwise scatterplots between Factors 1, 4, 8 and 10, with colors denoting HAP and no HAP. Boxplots show the median (black horizontal line) and the first and third quartiles (ends of the box). (D) Temporal kinetics within the HAP groups as captured by each factor value. Lines are smoothed over time using the respective group medians, with shaded zones indicating the standard error. Statistical significance was assessed (B) with *t* test and (C) with Wilcoxon rank-sum test. ∗∗∗*p* < 0.001 and ∗∗∗∗*p* < 0.0001.

To uncover the temporal patterns and the main driving forces of the alterations of the host-lung microbiome interactions, we implemented MEFISTO (Modeling Environmental Factors and their Interactions with Stochastic Outcomes) (Figure 2A).^18^ MEFISTO is an unsupervised artificial intelligence pipeline that analyzes multi-omics data across different conditions and time points. It integrates omics data to identify key patterns and interactions (called factors) that characterize omics data evolution over time. We tested the associations of 15 multi-omics factors with clinical variables, identifying multiple factors associated with distinct clinical traits (Figure 2B; Figure S8A). Several multi-omics factors showed associations with specific clinical variables. In particular, Factors 5 and 10 were associated with the duration of mechanical ventilation, while Factors 4 and 6 were associated with mortality, and Factor 1, despite being largely driven by the respiratory microbiome, was not associated with HAP. After verifying the data normalization and transformation process (Figure S8B), we questioned the role of the level of microbiome analyses (Figure S8C) in these results. Because phylum-level aggregation reduced microbiome-associated explained variance, whereas class, family, genus, and operational taxonomic unit representations gave broadly similar variance structures, we retained genus-level taxonomy as an interpretable compromise between coarse aggregation and increased sparsity.

Among the 15 factors, Factors 4 and 10 emerged as the most consistently associated with severe respiratory outcomes, including hemagglutinin, prolonged mechanical ventilation, and mortality and were therefore prioritized for downstream analyses. Smoking status, but not chronic obstructive lung disease, was negatively correlated with Factors 4 and 10, suggesting that these factors capture smoking-associated alterations rather than effects attributable to established chronic lung disease. Highlighting the significance of Factors 4 and 10 in the pathogenesis of respiratory complications and complicated in-ICU outcomes, we observed that pairwise combinations of Factors 4 and 10 enabled the identification of all longitudinal samples, including those collected during HAP (Figure 2C). Finally, the time courses for Factors 4 and 10 differed by HAP status, with lower factor levels in SIRS patients with HAP or HAP-induced ARDS (Figure 2D). In summary, by combining 5 omics and considering their longitudinal evolution to understand the immune-respiratory microbiome metasystem evolution during SIRS, we identified one multi-omic factor (Factor 4) that correlated with the development of HAP, duration of mechanical ventilation, and survival in patients with SIRS.

### Identifying inter-individual host-microbiome variations associated with respiratory complications

**Figure 3:**
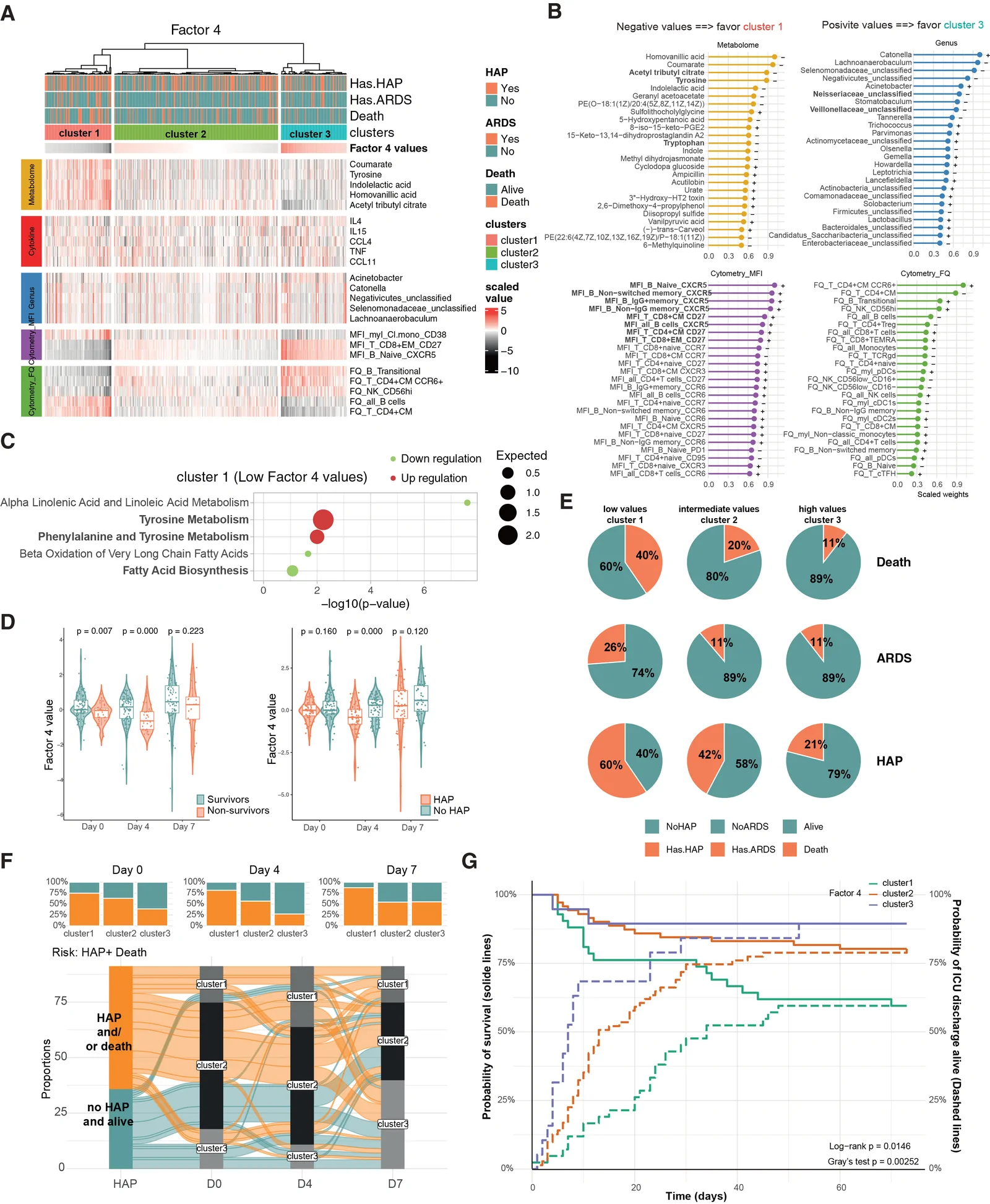
Multi-omic signature associated with severe respiratory complications in patients with SIRS (A) Unsupervised *k*-means clustering of Factor 4 values and top markers per omic in critically ill patients with top 5 features contributing to Factor 4 from each per-omic layer. (B) Top 25 features contributing to low (negative) or high (positive) Factor 4 values. (C) Increased and decreased metabolite sets in samples of cluster 1 from Factor 4. (D) Violin plots showing media, 25^th^–75^th^ percentiles, and min-max Factor 4 values in survivors vs. non-survivors and with or without HAP. (E) Numbers and percentages of survivors and patients with HAP or ARDS in samples by Factor 4 clusters. (F) Over-time movements between Factor 4 clusters. The histograms on the top show the proportions of patient within clusters. (G) Cumulative incidence curves for ICU discharge alive and survival curves in patients stratified by FFT score for host-microbiome endotype severity. Statistical significance was assessed by (C) hypergeometric test, (D) Wilcoxon rank-sum test, and (G) the log rank for the survival curves and Gray’s test for the cumulative incidence function (discharged alive) curves.

Factor 4 captured a large extent of inter-patient variance in the host-lung microbiome metasystem evolution and explained most of the inter-patient variance in the clinical view (complicated outcome). Thus, we focused our investigation on the association between inter-individual variations in Factor 4 and HAP. Using an unsupervised consensus *k*-means clustering approach, we defined three sample clusters with increasing Factor 4 values (Figure 3A). The primary sources of Factor 4 variance were the blood metabolome, leukocyte phenotypes, and respiratory microbiome (Figures 3A and 3B). Among the main blood metabolic features contributing to low Factor 4 values, we identified elevated acetyl tributyl citrate, tyrosine, and tryptophan levels (Figures 3B; Figure S9C). Better describing the pathophysiological process, the pathway analysis showed that the metabolites associated with low Factor 4 values (cluster 1) were characterized by increased metabolism of tyrosine, which participates in hormone and neurotransmitter syntheses (including epinephrine)^19^ and whose metabolism by the microbiome regulates B-cell-dependent lung inflammation during infections^20^^,^^21^^,^^22^ (Figure 3C), and decreased fatty acid biosynthesis, which can be mediated by a healthy microbiome.^23^^,^^24^

In the immune landscape, high expression of CXCR5 on B cells, a chemokine receptor critical for homing to lymphoid organs and facilitating B cell-T follicular helper cell colocalization,^25^ and of CCR6, which directs B cells to mucosal and inflamed tissues,^26^ correlated with elevated Factor 4 scores. Similarly, CD27 expression on CD4^+^, CD8^+^, and γδ T cells,^27^ which enhances T cell receptor-mediated activation, was also associated with high Factor 4 values (Figures 3A and 3B; Figure S9B). On the contrary, CD38, an enzyme that mediates cell adhesion and migration by metabolizing NAD^+^, was expressed on monocytes and was associated with low Factor 4 values. Regarding the respiratory microbiome, anaerobic genera, usually identified in the oropharynx rather than in the distal airways, such as of the family Neisseriaceae,^14^ also contributed to low Factor 4 values (cluster 1, Figures 3A and 3B).

We then examined whether the individual variation of this multi-omic factor was clinically meaningful. We found that Factor 4 values were higher in survivors than in non-survivors upon ICU admission and on day 4, and in patients without HAP at day 4 (Figure 3D). At each time point, the rates of HAP, ARDS, and death were increased in cluster 1 (low Factor 4 values) samples (Figure 3E). Figure 3F shows the evolution of clusters over time. Patients with favorable outcomes, defined as survivors without HAP, remained in clusters 2 (medium Factor 4 values) or 3 (high Factor 4 values) and never in cluster 1 (low Factor 4 values). Finally, we found that the probabilities of survival and of being discharged alive from the ICU were decreased in patients in cluster 1 (Figure 3G and Figure S9D for the probability of being discharged alive from mechanical ventilation). In this series of analyses, we have identified associations between B cell differentiation and trafficking, anaerobic bacteria, and high tyrosine metabolism, as well as low fatty acid biosynthesis. Significantly, the inter-individual variability of this metacluster was associated with vital clinical outcomes, including the duration of life support need and survival.

### A simplified signature for assessing the severity of host-microbiome interaction alterations

**Figure 4:**
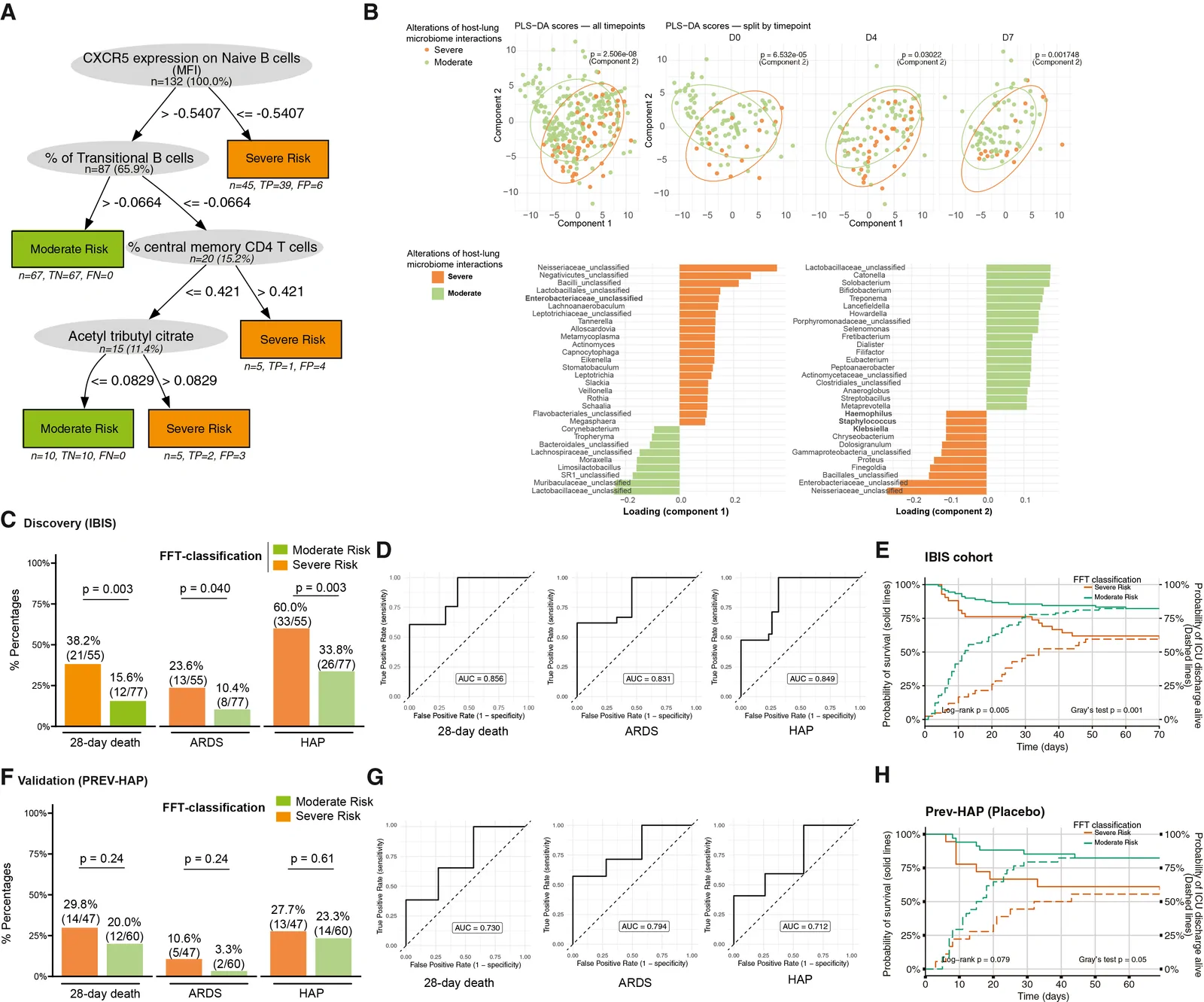
Association of host-microbiome endotype severity with unfavorable outcomes (A) Fast-and-frugal tree (FFT) multi-omic staging scheme to predict Factor 4 clusters stratified into high- and moderate-risk classes. (B) PLS-DA-based comparison of the respiratory microbiomes of ETA samples from patients in the IBIS cohort, showing projections onto the first two latent components (C1 and C2) across all samples and by time point (day [D] 0, D4, and D7), with the top 30 taxa contributing to each component. Ellipses represent 95% confidence intervals. (C) Bars showing percentages of HAP, ARDS, and 28-day death in moderate vs. severe host-microbiome endotype in the discovery cohort (IBIS). (D) Receiver operating characteristic (ROC) of FFT for HAP, ARDS, and death in the discovery cohort. (E) Cumulative incidence curves for ICU discharge alive and survival curves in patients stratified by FFT score for host-microbiome endotype severity. (F–H) Same analyses repeated in the validation cohort (PREV-HAP randomized clinical trial). Statistical significance was assessed by (B) Wilcoxon test on component 2 of the PLS-DA, (C and F) the chi-squared test, and (E and H) the log rank for the survival curves and Gray’s test for the cumulative incidence function (discharged alive placebo group) curves.

These endotypes were derived from longitudinal modeling of multiple metabolites, immunological assays, and respiratory microbiome profiles, which limits their applicability for patient classification and does not enable external validation in an independent cohort. Therefore, we employed a stacked machine learning model to define a parsimonious classification scheme using a minimal number of criteria. Fast-and-frugal tree (FFT)-based modeling offered a simple, 4-factor decision tree that individually classifies samples as severe (cluster 1) vs. moderate alterations of the host-lung microbiome interactions (clusters 2–3) based on CXCR5 membrane expression on B cells, frequencies of transitional B cells and central memory CD4 T cells, and blood levels of acetyl tributyl citrate (Figure 4A). We applied a 5-fold cross-validation scheme to assess the model’s accuracy for cluster 1 identification and calculated a mean classification area under the curve of 0.951 (Figure S10A).

Partial least squares discriminant analysis (PLS-DA) of respiratory microbiome profiles showed a statistically significant yet partially overlapping separation between patients classified as having severe or moderate alterations in host-microbiome interactions (Figure 4B). This indicates that the simplified score is associated with limited differences in respiratory microbiomes after day 3. We confirmed that this score reasonably predicts 28-day death, ARDS, and HAP in the discovery cohort (Figures 4C and 4D). Moreover, patients with a severe alteration signature tended to have lower probabilities of survival and of being discharged alive from the ICU than patients with moderate alterations of host-lung microbiome interactions (Figure 4E). In the PREV-HAP independent validation cohort, the severe alteration signature showed directionally consistent trends and moderate discriminatory performance (Figures 4F and 4G), with the clearest time-to-event separation observed in the placebo subgroup, where treatment-related heterogeneity was absent (Figure 4H).

Because antibiotic exposure could contribute to host-lung microbiome alterations captured by the multi-omic analysis, we also analyzed antibiotic exposure across the severity classes of host-microbiome interaction alterations identified in the IBIS cohort. First, the rates of patients receiving antimicrobial therapy increased over time in patients with severe alterations, but without a significant difference with patients with moderate alterations (Figure S10B). Moreover, antibiotic use can reduce alpha diversity in the microbiome by indiscriminately killing both the pathogen and commensal bacteria, disrupting the balance and richness of microbial species in the ecosystem. However, the alpha diversity (Shanon index) was not statistically different between moderate and severe alterations samples (Figures S9B and S10C for the discovery and validation cohorts, respectively), arguing against an essential role of antimicrobial therapy.

### Host-lung microbiome endotype severity for the identification of responders and non-responders to interferon-gamma treatment

Since PREV-HAP patients were randomly assigned to IFN-γ treatment or placebo, we questioned if the accuracy of this interaction signature for patient outcome prediction was altered in the IFN-γ treatment group. In the placebo group, 18 (34.6%) patients had severe alterations in host-lung microbiome interactions and 34 (65.4%) had moderate alterations. The accuracies of the FFT score for 28-day death, ARDS, and HAP were 0.789, 1.00, and 0.761, respectively (Figure S11A and S11B). The probability of being discharged alive from the ICU was lower in patients in the placebo group who had severe alteration than in those who had moderate alterations (Figure S11C). At the same time, it was not different in patients from the IFN-γ group who had severe alterations and those who had moderate alterations (Figure S11D). These results suggested an interaction between the response to IFN-γ treatment and the severity of alterations in host-lung microbiome interactions.

**Figure 5:**
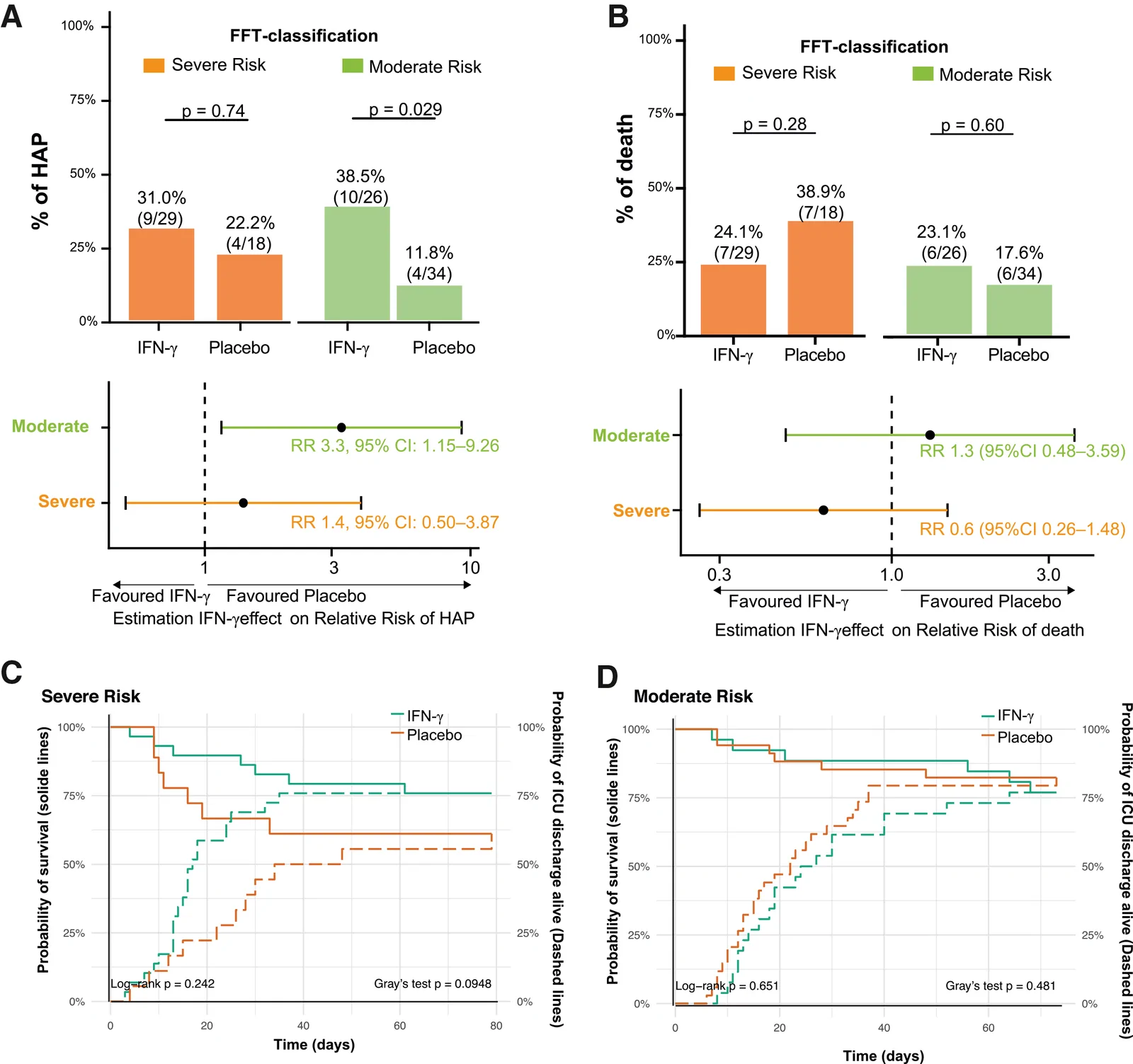
Effects of IFN-γ treatment on HAP and 28-day death according to host-microbiome endotype severity scores (A) Bars showing percentages of HAP and forest plots showing RR (95% CI) of HAP with IFN-γ treatment in moderate vs. severe host-microbiome endotype. (B) Bars showing percentages of 28-day mortality and forest plots showing RR (95% CI) of 28-day death with IFN-γ treatment in moderate vs. severe host-microbiome endotype. (C and D) (C) Cumulative incidence curves for ICU discharge alive and survival curves in patients with severe or (D) moderate host-microbiome endotype treated with IFN-γ treatment or placebo score. Statistical significance was assessed by (A and B) the chi-squared test and (C and D) the log rank for the survival curves and the Gray’s test for the cumulative incidence function (discharged alive) curves.

Finally, we tested in an exploratory analysis whether the IFN-γ treatment effects varied with the severity of host-microbiome interaction alterations. The risk of HAP was increased by IFN-γ treatment in patients with moderate signature (risk ratio [RR], 3.3; 95% CI, 1.15–9.26; *p* = 0.029), but not in those with severe signature (RR, 1.4; 95% CI, 0.50–3.87; *p* = 0.74; Figure 5A). Also, we observed that the relative risk of death with IFN-γ treatment was 0.6 (95% CI, 0.26–1.48; *p* = 0.28) in patients with severe alterations and 1.30 (95% CI, 0.48–3.59; *p* = 0.60) in those with moderate alterations (Figure 5B). Finally, IFN-γ treatment tended to increase the probability of survival and of discharge alive from the ICU in patients with severe alterations (Figure 5C) and had no apparent effect on these outcomes in patients with moderate alterations (Figure 5D), suggesting an interaction between IFN-γ treatment effects and the severity classification.

## Discussion

Here, we report a longitudinal exploration and comprehensive analysis of host-respiratory microbiome interactions in critically ill humans with SIRS. The temporal analysis of respiratory microbiome sequencing, flow cytometry, cytokinome, and metabolome data demonstrated that the respiratory microbiome and circulating immune cells form a dynamic metasystem with which tyrosine metabolism and fatty acid biosynthesis are associated. The development and external validation of a simplified signature for individual classification of host-lung microbiome interaction alterations also showed that these factors are associated with the response to IFN-γ treatment.

The classical understanding of pneumonia pathophysiology is that lung infection with pathogens activates innate immunity and induces inflammation-mediated lung injury. The role of the lung microbiome, composed of non-pathogenic bacteria, viruses, and fungi, has attracted interest in respiratory health^28^^,^ as alterations in its composition, independent of the presence or absence of pathogens, are associated with immune cell transcriptomic activity and pneumonia severity.^14^^,^^29^ However, while studying only the respiratory microbiota composition failed to discriminate between community-acquired pneumonia and uninfected humans accurately,^30^ the combination of host and microbiome compositions enables the fair identification of critically ill patients with or without lower respiratory tract infections.^28^ Thus, we and others have proposed the concept of lung dysbiosis, defined as alterations of host-microbiome interactions, as the main pathophysiological mechanisms regulating pneumonia severity and response to treatment.^13^ In this study, the identified moderate and severe states does not represent microbial overgrowth alone but a systemic immune-metabolic state reflected at the airway interface. The respiratory microbiome appears embedded within broader host inflammatory reprogramming, such that microbial shifts mirror and potentially amplify systemic immune alterations. Severe alteration of host-lung microbiome interactions may, therefore, represent a vulnerability phenotype characterized by reduced host ecological resilience rather than simply a change in microbial composition. This study provides additional evidence for this concept by revealing the existence of metaclusters composed of lung bacterial genera and metabolites, as well as leukocyte subsets, whose modulation during SIRS is associated with pneumonia severity, thereby strengthening the role for altered host-microbiome interactions in the pathophysiology of this dramatic condition. This gained knowledge also informs us about the causes of the rapid alterations in the respiratory microbiome composition observed in patients with SIRS.

The crosstalk between the gut microbiome and systemic immunity has been described in numerous human medical conditions, including sepsis^10^ and cancer.^31^^,^^32^ Experimental mouse models have proposed that the mechanisms enabling the control of remote organs by gut microbiome are as diverse as the translocation of commensal bacteria,^33^ the systemic release of bacterial-derived metabolites such as short fatty acids,^34^ or the systemic circulation of leukocytes trained in the gut.^35^ Given the lower biomass of the lung microbiome, the possibility of lung commensal bacteria regulating systemic immunity has remained a matter of debate. In contrast to the gut microbiome, whose contribution to systemic inflammation in critical illness is often attributed to high microbial biomass and barrier disruption leading to translocation of microbial products, the lung microbiome has substantially lower biomass, making direct microbial translocation an unlikely dominant mechanism.^36^ Our findings, instead, support a framework in which the lung microbiome influences systemic inflammation indirectly, through modulation of local immune activation and metabolic programs that are reflected in circulating immune and metabolomic profiles. In this view, the lung microbiome acts as a regional immune modulator whose effects are amplified through host immune and metabolic networks rather than through microbial burden alone. Recently, the lung microbiome was shown to regulate brain inflammation in a mouse model of autoimmunity, but the mechanism was incompletely understood.^37^ Here, our results showed that alterations in systemic conditions, blood metabolites, and immune cell trafficking are associated with modifications in the respiratory microbiome. Significantly, while most bacterial genera-metabolite correlations changed over time, only a fraction was related to the development of severe respiratory complications. Our results suggest that tyrosine metabolism and fatty acid biosynthesis are essential communication pathways between the lung microbiome and immunity in critically ill humans with SIRS.

Our results enable the identification of a metacluster of host-microbiome interactions whose expression levels are associated with unfavorable outcomes. Throughout this study, host-microbiome interaction metaclusters refer to a coordinated multi-omic state integrating respiratory microbiome composition with circulating immune and metabolomic alterations, rather than a microbiome-only ecological measure. Accordingly, the terms moderate and severe denote the magnitude and clinical association of this integrated host-lung microbiome state and are not intended to reflect the alpha diversity or other standalone microbiome ecology indices. These severe alterations of the host-lung microbiome interactions were characterized by increased metabolic pathways beneficial to both the microbiome and the host, such as the tyrosine pathway. Hyperactivation of the tyrosine kinase pathway was associated with reduced phagocytic capacity of resident alveolar macrophages during severe pneumonia and with participation in endogenous catecholamine production during sepsis.^19^^,^^38^^,^^39^ Beyond its role as a catecholamine precursor, tyrosine metabolism is tightly linked to immune cell activation, redox balance, and macrophage functional programming. Increased reliance on tyrosine-related pathways during SIRS may, therefore, reflect a shift toward stress-adaptive immune metabolism that could impair effective microbial clearance. In this context, metabolic reprogramming and microbial community shifts may arise as coupled consequences of systemic inflammatory stress. We also found that reductions in fatty acid biosynthesis and β-oxidation of very long chain fatty acid-related pathways were associated with severe host lung microbiome dysbiosis. This enrichment was driven by a limited number of annotated fatty acids detected in the untargeted metabolomic dataset, including dodecanoic acid and hexacosanoic acid, rather than by direct quantification of a broad range of very-long-chain fatty acids or complex lipid subclasses. Fatty acid oxidation becomes an important energy source during acute inflammation, such as during sepsis.^40^ Reduced fatty acid biosynthesis and oxidation may reflect impaired metabolic flexibility of immune cells during systemic inflammation. Such metabolic constraints could limit antimicrobial effector functions and thereby weaken the host control of airway microbial communities. This observation could provide a rationale for the association between blood lipid alterations and HAP in patients with COVID-19.^41^ Finally, the role of the immune-lipid-microbe pathway was also in the gut during acute inflammation,^9^ suggesting the existence of common regulatory pathways between the gut and the respiratory microbiome in patients with SIRS.

While elevated IFN-γ levels in the blood characterize SIRS, decreased IFN-γ production during treatment was also associated with greater critical illness severity and unfavorable outcomes.^42^^,^^43^ So, the dual role of IFN-γ in patients with SIRS, potentially reducing the risk of secondary infection or increasing the risk of inflammation-induced secondary organ failure, is still incompletely understood. IFN-γ levels in the blood were not identified in features participating in the Factor 4 metacluster; however, an exploratory analysis identified an interaction between IFN-γ treatment effects and host-lung microbiome metacluster alterations in patients with SIRS. IFN-γ treatment restored the glycolytic/oxidative balance of exhausted lymphocytes in septic patients^44^ and had broad metabolic effects.^45^ We interpreted these results as indicating that IFN-γ endogenous production does belong to the identified host-lung microbiome metacluster, yet that IFN-γ treatment can favorably restore functions of pulmonary host-microbiome features. Although the present study is observational, it generates testable hypotheses regarding the role of host-lung microbiome interactions in respiratory complications. In particular, approaches aimed at maintaining or restoring a symbiotic lung microbiome, rather than eradicating microbial communities, may clarify whether the host-lung microbiome metacluster states identified here act as drivers of disease progression or markers of host response.

This study has several limitations. First, interpretation of these findings should also consider limitations inherent to multi-omics integration. Latent factor models prioritize dominant sources of structured variation and may underrepresent weaker but biologically relevant signals, particularly in sparse data layers such as the respiratory microbiome. In addition, the relative contribution of each omic layer reflects both biological variance and technical characteristics. Second, the validation cohort included patients enrolled in an IFN-γ prevention trial, with only a subset receiving the treatment. While this may restrict the generalizability of the classification to entirely untreated populations, it also enabled the identification of IFN-γ-related mechanisms linking host and microbiome features. Finally in the validation cohort, patients were stratified according to host-microbiome severity using samples collected at day 3–4 after initiation of interferon-gamma or placebo treatment. Consequently, the observed associations with clinical outcomes reflect early-on-treatment host-microbiome interaction states rather than baseline states present before treatment.

Several studies have demonstrated that critical illnesses induce profound alterations in the immune system and microbiome compositions. Our study completed these observations by showing that the respiratory microbiome, circulating immune cells, blood cytokines, and metabolites form dynamic and functional metaclusters. Thus, longitudinal modeling of host-lung microbiome interactions enabled the discovery of actionable pathophysiological mechanisms underlying severe respiratory complications and validated a robust signature associated with the response to interferon-gamma in critically ill patients.

### Limitations of the study

While our study suggests that this multi-omic score can be used to identify responders and non-responders to interferon-γ treatment, the validation of this proposition in a randomized clinical trial is required before implementing this result in daily cares.

## Resource availability

### Lead contact

Further information and requests for resources and reagents should be directed to and will be fulfilled by the lead contact, Antoine Roquilly (antoine.roquilly@univ-nantes.fr).

### Materials availability

This study did not generate new unique reagents.

### Data and code availability

Respiratory microbiome sequencing data supporting the findings of this study have been deposited in the NCBI Sequence Read Archive under accession code PRJNA1109383. Processed/derived metabolomics feature tables used in this study have been deposited in Zenodo under https://doi.org/10.5281/zenodo.18768243. Filtered microbiome, cytometry MFI, cytometry frequency, cytokine, metabolomics, and associated clinical metadata matrices used for MEFISTO analysis are also available in Zenodo under https://doi.org/10.5281/zenodo.18768243. All other data reported in this paper are available in the main text and supplementary information. The full analysis code used in this study has been deposited on Zenodo at https://doi.org/10.5281/zenodo.18768243. Any additional information required to reanalyze the data reported in this work paper is available from the lead contact upon request.

## Acknowledgments

We thank the biological resource center for biobanking of CHU Nantes (Hôtel Dieu, Center de Ressources Biologiques, Nantes, F-44093, France, BRIF: BB-0033-00040). We thank Mrs. Cecilia Le Bel, Noelline Mathelier, Mr. Flavien Counille, Maelle Ningre, and Aurelia Kuster (CHU Nantes) and Amelie Guisseau (AP/HP) for technical and administrative assistance. Study funder: European Union’s Horizon 2020 research and innovation program under grant agreement number 847782 (HAP2 project). Funding: A.R. received a grant from the European Union’s Horizon 2020 research and innovation program under grant agreement number 847782, the Agence National de la Recherche (ANR) PROGRAM project, and the MSD Avenir grant (Phenomenon project).

## Author contributions

D.S., M.P., J.P., and A.R. selected studies, performed data analyses, interpreted results, and drafted the manuscript. F.P.M., C.P., D.F.D., D.K., L.F.-B., and A.T. included patients, managed samples, collected clinical data, and revised the manuscript. S.K., C.B., and M.G. performed data analyses, interpreted results, and revised the manuscript. B.B., M.G.N., R.P.D., E.M., and J.P. interpreted the results and revised the manuscript. All authors have approved the final manuscript for publication.

## Declaration of interests

The authors declare no competing interests.

## STAR★Methods

### Key resources table

**Table:** 

REAGENT or RESOURCE	SOURCE	IDENTIFIER
**Antibodies**		

LIVE/DEAD Fixable Blue Dead Cell Stain Kit	Thermo Scientific	N/A
Human TruStain FcX Blocker	BioLegend	N/A
True-Stain Monocyte Blocker	BioLegend	N/A
Transcription Factor Staining Buffer Set	eBioscience	N/A
Cytofix/Cytoperm reagent	BD Biosciences	N/A
Pre-conjugated anti-human flow cytometric antibodies against chemokine receptors and other surface markers, with intracellular labeling	BioLegend; BD Biosciences; Thermo Fisher Scientific; eBioscience	see Tables S2, S3, and S4

**Chemicals, peptides, and recombinant proteins**		

Phorbol 12-myristate 13-acetate	Sigma-Aldrich	N/A
Ionomycin	Sigma-Aldrich	N/A
3-amino-1-propanesulfonic acid	Sigma-Aldrich	N/A
Brefeldin A	BD Biosciences	N/A
Monensin	BD Biosciences	N/A
Resiquimod (R848)	InvivoGen	N/A
Hexakis(1H,1H,3H-tetrafluoropropoxy)-phosphazene	Agilent	N/A

**Critical commercial assays**		

Luminex Procartaplex 34-plex cytokine immunoassay panel	Thermo Fisher Scientific	Ref# EPXR340-12167-901
Olink® Explore 384 Inflammation panel	Olink Proteomics AB	Explore 384 Inflammation
Qiagen DNeasy Blood & Tissue Kit	Qiagen	N/A
MiSeq Reagent Kit v2 (500 cycles)	Illumina	N/A

**Deposited data**		

16S rRNA gene sequencing data	NCBI Sequence Read Archive (SRA)	PRJNA1109383
Metabolome processed data	Excel file deposited in Zenodo	https://doi.org/10.5281/zenodo.18768243
Processed deidentified multi-omic data matrices and clinical metadata	CSV files deposited in Zenodo	https://doi.org/10.5281/zenodo.18768243
Full analysis code	R codes deposited in Zenodo	https://doi.org/10.5281/zenodo.18768243

**Oligonucleotides**		

16S rRNA gene primer 63F	Previously published protocol (Schloss lab)	5′-GCAGGCCTAACACATGCAAGTC-3′
16S rRNA gene primer 355R	Previously published protocol (Schloss lab)	5′-CTGCTGCCTCCCGTAGGAGT-3′

**Software and algorithms**		

R statistical software	R Foundation for Statistical Computing	Version 4.4.1
Python	Anaconda Distribution	Version 3.12.4
FlowJo	Tree Star Inc.	Version 10.8.1
Quantisoft software	Bio-Rad	N/A
xPONENT software	Luminex	Version 4.2
Procartaplex Analyst	Thermo Fisher Scientific	Version 1.0
MEFISTO (MOFA2 framework)	MOFA2 R package	Version 1.14.0
mixKernel	mixOmics R package	Version 0.9–1
mixOmics	R package	Version 6.28.0
MetaboAnalystR	R package	Version 4.0.0
FFTrees	R package	Version 2.0.0
ConsensusClusterPlus	R package	Version 1.68.0

**Other**		

Agilent 6550 iFunnel LC-MS Q-TOF mass spectrometer	Agilent Technologies	N/A
Agilent 1260 Infinity II quaternary pump	Agilent Technologies	N/A
MPS3 autosampler	Gerstel	N/A
Illumina MiSeq platform	Illumina	N/A
Cytek Aurora spectral flow cytometer	Cytek Biosciences	N/A
QX200 Droplet Digital PCR System	Bio-Rad	N/A
C1000 Touch Thermal Cycler	Bio-Rad	N/A

### Experimental model and study participant details

#### Human participants – Discovery cohort

Patients were enrolled from 31st January 2017 to 5th May 2020 in two French Surgical Intensive Care Units at a university hospital (Nantes, France) [14,16]. The collection of human samples has been declared to the French Ministry of Health (Program de recherche “Immunologie”, DC-2017-2987), and was approved by the Comite de Protection des Personnes Ouest IV (7/04/2015 and 08/10/2020). Written informed consent from a next-of-kin was required for enrollment. Retrospective consent was obtained from patients when possible. Appropriate consent was obtained for the release of information from deceased individuals. Participants received no compensation. Inclusion criteria were male or female, 18–80 years old, brain injury (Glasgow Coma Scale below or equal to 12 and abnormal brain-CT scan) and receiving invasive mechanical ventilation. Exclusion criteria were immunosuppressive drugs, and pregnancy. A total of 157 patients with available clinical and biological data were included in the discovery cohort. Baseline demographic and clinical characteristics, including age and sex, are reported in Table S1. All patients were clinically followed up for 90 days. HAP and ARDS diagnoses were blindly reviewed by intensivists for compliance with international definitions 43–45. Blood and endotracheal aspirates were collected at day 0 (<48 h after ICU admission, day 3–4 and day 7–8). Plasma and endotracheal aspirates were immediately frozen at −80c, and stored until sequencing. Fresh PBMCs were isolated, placed in 4%-albumin and 10%DMSO, and then frozen in liquid nitrogen until analyses.

#### Human participants – Validation cohort

The PREV-HAP trial was an investigator-initiated multicenter, parallel-group, double-blind, randomized clinical trial performed in 9 French ICUs 17. The Ouest II Angers (France) Ethics Committee approved the study protocol in March 2021. This trial complied with the Declaration of Helsinki and was registered in March 2021 (number clinicaltrial.gov NCT04793568). The patient’s legal surrogate provided written informed consent for participation. In agreement with the local laws, patients could be enrolled before the provision of legal surrogate consent if the next of kin could not be informed within the maximum delay for inclusion. When able to provide it, patient’s follow-up consent was requested up to 90 days after inclusion. Patients aged between 18 and 85 years, receiving invasive mechanical ventilation, with one or more acute organ failures at the time of inclusion were randomized to interferon gamma-1b (1b (100 μg/0.5 mL vials, IMUKIN, from Clinigen) or placebo (normal saline vials) in fixed blocks of 6, in a 1:1 ratio, with stratification based on the cause of hospitalization (sepsis vs. other) and the country (France, Greece, Spain). Participants received five subcutaneous injections of 100 μg of recombinant interferon gamma-1b (interferon-gamma group) or matching placebo (placebo group) from day 1 to day 9 (i.e., one injection every 48h). A total of 108 patients with available clinical and biological data were included in the validation cohort. Baseline demographic and clinical characteristics, including age and sex, are reported in Table S1. Patients were followed-up for 90 days, and HAP and ARDS diagnoses were blindly reviewed by intensivists for compliance with international definitions 43–45. Blood and endotracheal aspirates were collected at day 0 (before the first study treatment injection), days 3–4 and 7–8. Plasma and endotracheal aspirates were immediately frozen at −80c, and stored until sequencing. Fresh PBMCs were isolated and then frozen in liquid nitrogen until analyses.

Both male and female participants were eligible for inclusion in both cohorts. The study was not powered to assess the independent influence of sex or gender on multi-omic profiles or clinical outcomes, and these associations were therefore not analyzed as primary endpoints.

### Method details

#### Plasma metabolomics profiling

Plasma samples were stored at −80°C and thawed on ice prior to analysis. For each sample, 20 μL of plasma were transferred to a 96-deepwell plate and extracted by adding 180 μL of a chilled solution of 80% methanol in water (v/v). Plates were capped with a silicone cap mat, mixed for 15 s, incubated for 1 h at 4°C, and centrifuged for 30 min at 3,750 rpm at 4°C. One hundred microliters of the supernatant were transferred to new 96-well plates and diluted 1:10 or 1:100 in 80% methanol (v/v) for instrument analysis.

A pooled study sample (pSS) was prepared by pooling 5 μL of each extract and was injected periodically throughout the batch to monitor analytical stability. Samples were acquired in randomized order within plates.

Metabolomic profiles were acquired using flow-injection mass spectrometry on an Agilent 6550 iFunnel LC-MS Q-TOF mass spectrometer coupled to an MPS3 autosampler (Gerstel) and an Agilent 1260 Infinity II quaternary pump. The running buffer consisted of 60% isopropanol in water (v/v) buffered with 1 mM ammonium fluoride. Hexakis(1H,1H,3H-tetrafluoropropoxy)-phosphazene and 3-amino-1-propanesulfonic acid were added to the running buffer as lock masses. The instrument was operated in 4 GHz high-resolution negative ionization mode, with mass spectra acquired between 50 and 1,000 m/z in profile mode. Five microliters of each sample were injected twice consecutively within 0.96 min to serve as technical replicates.

Raw mass spectral profile data were centroided, merged, and recalibrated using a flow-injection mass spectrometry preprocessing pipeline adapted from Fuhrer et al. (2011), implemented in MATLAB. Putative metabolite annotations were generated using accurate mass-to-charge ratios (tolerance 0.001 m/z) and isotopic correlation patterns by matching against the Human Metabolome Database (HMDB), KEGG, and ChEBI databases.

For downstream analyses, technical replicates were aggregated at the feature level to generate a metabolite-by-sample intensity matrix. Prior to statistical analysis and multi-omic integration, metabolite intensities were log-transformed where appropriate to reduce right-skewness and standardized across samples.

#### Plasma cytokine quantification

In the discovery cohort, the cytokine plasma levels were measured by Luminex 34-cytokines panel. In the validation cohort (randomised trial). Interferon-gamma and IL12B plasma levels were measured on frozen serum using Olink Explore 384 Inflammatory panels (Olink Proteomics AB, Uppsala, Sweden) according to the manufacturer’s instructions.

#### Respiratory microbiome profiling

**Bacterial DNA isolation** Tracheal aspirates were aliquoted and a maximum of 500 μL of specimen from each was used for DNA extraction. Exact volumes of each specimen were documented. Sterile laboratory water and AE buffer used in DNA isolation were collected and analyzed as potential sources of contamination, as were extraction controls (empty isolation tubes) and blank sequencing wells. Genomic DNA was extracted (Qiagen DNeasy Blood & Tissue kit, Qiagen, Hilden, Germany) using a modified protocol previously demonstrated to isolate bacterial DNA1. Specimens were processed in a randomized order to minimize the risk of false pattern formation due to reagent contamination.

**16s rRNA gene sequencing** The V4 region of the 16s rRNA gene was amplified using published primers3 and the dual-indexing sequencing strategy developed by the laboratory of Patrick D. Schloss4. Sequencing was performed using the Illumina MiSeq platform (San Diego, CA), using a MiSeq Reagent Kit V2 (500 cycles), according to the manufacturer’s instructions with modifications found in the Schloss SOP5. Accuprime High Fidelity Taq was used in place of Accuprime Pfx SuperMix. Primary PCR cycling conditions were 95°C for two minutes, followed by 20 cycles of touchdown PCR (95°C 20 s, 60°C 20 s and decreasing 0.3° each cycle, 72°C 5 min), then 20 cycles of standard PCR (95°C for 20 s, 55°C for 15 s, and 72°C for 5 min), and finished with 72°C for 10 min.

**Bacterial DNA quantification** Bacterial DNA was quantified using a QX200 Droplet Digital PCR System (BioRad, Hercules, CA). Primers and cycling conditions were performed according to a previously published protocol6. Specifically, primers were 5′- GCAGGCCTAACACATGCAAGTC-3’ (63F) and 5′- CTGCTGCCTCCCGTAGGAGT-3’ (355R). The cycling protocol was 1 cycle at 95°C for 5 min, 40 cycles at 95°C for 15 s and 60°C for 1 min, 1 cycle at 4°C for 5 min, and 1 cycle at 90°C for 5 min all at a ramp rate of 2°C/s. The BioRad C1000 Touch Thermal Cycler was used for PCR cycling. Droplets were quantified using the Bio-Rad Quantisoft software. Two replicates were used per sample. No-template control specimens were used and were run alongside mini-BAL specimens.

**Generation of OTU/genus tables and statistical analysis** Sequence data were processed and analyzed using the software mothur according to the Standard Operating Procedure for MiSeq sequence data using a minimum sequence length of 250 basepairs. For each experiment and sequencing run, a shared community file and a phylotyped (genus-level grouping) file were generated using operational taxonomic units (OTUs) binned at 97% identity generated using the dist.seqs, cluster, make.shared and classify.otu commands in mothur. OTU numbers were arbitrarily assigned in the binning process and are referred to throughout the manuscript in association with their most specified level of taxonomy. Classification of OTUs was carried out using the mothur implementation of the Ribosomal Database Project (RDP) Classifier and the RDP taxonomy training set 14 (Trainset14_032015.rdp), available on the mothur website. We performed microbial ecology analysis using the vegan package and mvabund in R8-10. For relative abundance and ordination analysis, samples were normalized to the percent of total reads, and we restricted analysis to OTUs that were present at greater than 1% of the sample population. All OTUs were included in diversity analysis. Direct community similarity comparisons were performed using the Bray-Curtis similarity index. We performed ordinations using Principal Component Analysis on Hellinger-transformed normalized OTU tables generated using Euclidean distances. We determined significance of differences in community composition using PERMANOVA with 10,000 permutations using Euclidean distances.

#### Flow cytometry acquisition

PBMCs were thawed using Cryo thaw devices (Medax). Briefly, cells were resuspended in cell culture medium supplemented with 2U ml^−1^ benzonase by centrifugation (300 r.cf.; 7 min; 24°C). Cell count was calculated using an automated cell counter (Bio-Rad). Due to the resulting cell count, cells were used for all panels or surface panels only. Subsequent procedures, including short-term reactivation of cryopreserved PBMCs and cytometry analysis, were performed as described previously ^47^. Briefly, 2 million (mio) cells per sample were directly stained for cytometry analysis (surface panel), while 1 mio cells were restimulated with 50 ng mL^−1^ phorbol 12-myristate 13-acetate (Sigma–Aldrich) and 500 ng mL^−1^ ionomycin (Sigma–Aldrich) in the presence of 1× Brefeldin A and 1x Monensin (both BD Biosciences) for 5 h at 37°C, or in case of R848 stimulation, 2.5 mio cells using 2μg mL^−1^ R848 (Invivogen) in the presence of 1× Brefeldin A and 1x Monensin (both BD Biosciences) for 8 h at 37°C. Before surface labeling for spectral flow cytometry, cells were washed with PBS and then resuspended in 100μL of Live Dead Fixable Blue mixture (Thermo Scientific, 1:500) followed by a washing step. To avoid nonspecific binding, the samples were resuspended in 30 μL of True Stain FcX (BioLegend) and incubated for 10 min at 4°C. Anti-human flow cytometric antibodies were purchased pre-conjugated (Tables S2, S3 and S4). 70 μL of the first surface-antibody mixture was added and cells were incubated for 15 min at 37°C. After another washing step, the second surface-antibody staining step (100 μL) was performed for 15 min at 4°C. Then, fixation was performed using 150 μL of 2% PFA for 15 min at 4°C. For intracellular spectral cytometry, after surface-antibody labeling, cells were fixed and permeabilized using Cytofix Cytoperm reagent (BD Biosciences) for 30 min at 4°C. Intracellular labeling was then performed in 100 μL of 1x permeabilization buffer (Thermo Scientific) for 11 h (Lymphoid cytokine panel, Table S4) or 10 h (Myeloid cytokine panel, Table S5) at 4°C. Spectral cytometry samples were acquired on a Cytek Aurora (Cytek Biosciences). Quality control of the Cytek Aurora was performed daily as instructed by the manufacturer. For downstream analysis, dead cells and doublets were excluded using FlowJo (TreeStar). Samples with viability lower than 10% and fewer than 500 live, CD45 positive cells were excluded. Cytometry data were transformed with an inverse hyperbolic sine (arcsinh) function using the R environment (range 30–18000). To balance the influence of markers with different dynamic ranges, we performed background subtraction and channel-based percentile normalisation using the 99.9th percentile of each marker across the whole dataset (Bendall et al., 2011). Pre-processed data were downsampled to a maximum of 150′000 cells per donor for the analysis of the main populations, all cells were used for analysis of the specific immune compartments. The high-dimensional analysis was performed using R, loosely based on a previously published workflow.^46^

#### Multi-omic integration (MEFISTO)

Five omics datasets were integrated using the MEFISTO framework with follow-up matching day as he temporal covariate 18. Each of these omics was represented as a unique modality or ‘view’ for input to MEFISTO. Samples available in only a subset of omics were also included in the input. Standard *Z* score normalization was performed on each omics input data matrix to resemble a Gaussian-like distribution (Figure S2B). Default parameters were used for model training. The final output resulted in the factorization of input into two major different kinds of matrices. The first matrix represented a single low-dimensional embedding of 15 latent Factors for every sample. Each MEFISTO factor captures a different dimension of heterogeneity in the data interpreted as analogous to the principal components in PCA. Mathematically, each factor ordinates samples along a one-dimensional axis centered at zero. Samples with different signs manifest opposite phenotypes along the inferred axis of variation, with higher absolute values indicating a stronger effect. MEFISTO also returned a ‘denoised” version of the input data with imputed values.

The second was a 2-dimensional weight matrix corresponding to each omics in the input. This weight matrix can be used to quantify the effect of an omics variable on a factor. The sign of the weight indicates the direction of the effect: a positive weight indicates that the variable has higher levels in the samples with positive factor values, and vice versa. Factor were stratified using consensus k-means clustering repeated 500 times (ComplexHeatmap in R) with optimal k set to 3 clusters.

#### Cluster differential analysis

Differential Analyses of Factor 4 clusters were performed on each omic to characterize unfavourable outcome-associated cluster (cluster 1). Using the MEFISTO model, the de-noised expression data from each omics was computed. These de-noised expression data from each omics were used to perform differential analysis between unfavourable outcome-associated cluster samples versus the rest pooled from other clusters. For the magnitude of difference between the two groups, the change in their median was measured.

#### Module score computation

Cluster-specific differential features were selected per omic by differential testing (padj <0.05 and |avgC| > 0) and their denoised feature values were extracted from the specified view. For each sample, the module score was computed as the median of significant upregulated features and visualized across clusters as boxplots.

#### Inter-omic kernel integration

Inter-omic concordance was evaluated using the mixKernel R package. Precomputed sample-similarity kernels from the metabolome, Luminex cytokine, microbiome, and cytometry (MFI and cell-frequency) datasets—derived from the same cohort and preprocessing inputs as the MEFISTO analysis—were block-scaled (scale = TRUE) and integrated through the cim.kernel function to compute kernel–kernel (RV-type) correlations. The resulting inter-block similarity matrix was visualized with corrplot.

#### Metabolite enrichment analysis

Over Representation Analysis (ORA) was performed on the differential metabolites between clusters. With the MetaboAnalystR package (version 4.0.0), an over-representation analysis was performed in known metabolite sets with at least 5 metabolites. These metabolites have been crossed with the Human Metabolome Database (HMDB) to identify enriched metabolic pathways.

#### Fast-and-frugal tree modeling

A fast-and-frugal decision tree (FFTree) classifier was trained using the **FFTrees** R package (version 2.0.0) to discriminate the positive class (“highRisk” = Cluster 1, Factor 4) from the rest. Trees were fit with the denoised data from the discovery cohort, optimizing the “goal” metric set to balanced accuracy. The best-performing tree on the training set—according to balanced accuracy—was selected, predictions were derived from that tree, and the corresponding balanced accuracy was reported. Receiver operating characteristic (ROC) curves were generated for the fast-and-frugal decision tree using class-membership probabilities taken from the exit (terminal) leaves of the best-performing tree.

#### External cohort validation

The external validation cohort underwent MEFISTO modeling to obtain denoised multi-omic data. These MEFISTO-derived features served as the test input for the trained FFTree model, which was applied without retraining to predict the day-4 (D4) risk outcomes. Model performance was evaluated by comparing the predicted high-risk assignments to the observed clinical outcomes in the validation cohort.

#### Survival and competing risks

Day-4 cohort records were analyzed with time measured either as days in the ICU (ICU analysis) or days on mechanical ventilation (ventilation analysis), with the x axis labeled accordingly. Input groups were defined by clusters from Factor 4. For the Kaplan–Meier (KM) component, death was the event; individuals who were alive were administratively right-censored at the maximum observed follow-up across subjects (i.e., deaths contributed their observed event time; all others were censored at the common horizon). KM curves were fit with the survival package (v3.7.0) and displayed as step functions anchored at probability 1 at time zero. For the competing-risks component, the cumulative incidence function (CIF) for “discharged alive” was estimated with cmprsk (v2.2.12) using standard cause coding (0 = censored, 1 = death, 2 = discharged alive, with cencode = 0); deaths were treated as the competing event, and subjects still hospitalized without discharge at last contact were censored. CIF estimates for the discharge-alive cause were retained, duplicates at identical times within groups were consolidated to the last available estimate, and curves were anchored at probability 0 at time zero. For each outcome (Has.HAP, Has.ARDS, Death), risk ratios (RR) were computed within predicted groups comparing IFNG versus Placebo, using a 0.5 continuity correction for zero counts. Ninety-five percent confidence intervals were derived on the log scale.

#### Low-dimensional projection

Partial least squares discriminant analysis (PLS-DA) was performed using the mixOmics R package on MOFA-denoised multi-omic data, with the risk group (highRisk vs. moderate) as the classification factor. Models were fit with two components and standardized features (scale = TRUE). Sample scores were visualized on the first two latent components, both jointly and stratified by timepoint, with 95% confidence ellipses per risk group. Top contributing features for each component were extracted from loading vectors and colored according to the risk class they favored.

#### Correlation trajectory analysis

Microbe–metabolite correlation trajectories were first identified in the discovery cohort (IBIS) using a baseline–maintenance rule that does not involve statistical testing. “Baseline interactions” were defined as pairs with an absolute correlation of |r_D0| ≥ 0.30 in either group. A pair was considered maintained at D4 or D7 if the correlation retained the same sign as baseline and the relative change from D0 did not exceed 20%; pairs failing this criterion at any post-baseline timepoint were labeled disrupted. Based on these rules, trajectories were classified into sets such as stable in HAP, disrupted in HAP, stable in NoHAP and disrupted in NoHAP.

### Quantification and statistical analysis

All statistical analyses were performed using R (R Foundation for Statistical Computing, Vienna, Austria). Specific package names and versions are indicated in the corresponding Method Details sections.

The exact value of n for each analysis is specified in the corresponding figure legends and results sections. Unless otherwise stated, n refers to the number of independent samples analyzed at the specified time point. For baseline characteristics, n refers to the number of patients.

Continuous variables are summarized as median and interquartile range (IQR). Categorical variables are summarized as counts and percentages.

For two-group comparisons of continuous variables, the Wilcoxon rank-sum test was used. Correlation analyses were performed using Spearman’s rank correlation coefficient.

For multi-feature differential analyses, *p*-values were adjusted for multiple testing using the Benjamini–Hochberg false discovery rate (FDR) procedure. A two-sided adjusted *p*-value <0.05 was considered statistically significant.

For contingency table analyses, a chi-square test without continuity correction was used when all expected cell counts were ≥5; otherwise, Fisher’s exact test was applied. Risk ratios (RR) were calculated using a 0.5 continuity correction for zero counts, and 95% confidence intervals were derived on the log scale.

For survival analyses, group differences were assessed by the log rank test for KM (via survminer v0.4.9, wrapping survdiff) and by Gray’s test from the cmprsk output for the discharge-alive CIF.

For correlation trajectory analyses, Fisher z–based tests were used to assess differences in correlation magnitude and temporal interaction effects. When applicable, *p*-values were derived using inverse-variance–weighted Z statistics.

No formal sample size calculation was performed for exploratory multi-omic analyses. Data were excluded only if predefined quality control criteria were not met, as described in the relevant Method Details sections.

Unless otherwise indicated, statistical significance is indicated as ∗*p* < 0.05, ∗∗*p* < 0.01, ∗∗∗*p* < 0.001, and ∗∗∗∗*p* < 0.0001. The statistical tests used for each analysis are specified in the corresponding figure legends.

#### ADDITIONAL RESOURCES

The PREV-HAP trial was registered at ClinicalTrials.gov under identifier NCT04793568.

## Supplemental information

Document S1. Figures S1–S11

Table S1. Baseline characteristics and clinical outcomes of patients in the IBIS and PREV-HAP cohorts Related to Figure 1A

Table S2. IBIS surface antibody panel for spectral flow cytometry Related to STAR Methods

Table S3. IBIS lymphoid intracellular cytokine antibody panel for spectral flow cytometry Related to STAR Methods

Table S4. IBIS myeloid intracellular cytokine antibody panel for spectral flow cytometry Related to STAR Methods

Table S5. PREV-HAP surface and intracellular antibody panel for spectral flow cytometry Related to STAR Methods

Table S6. PREV-HAP cytokine antibody panel for spectral flow cytometry Related to STAR Methods

Table S7. Human-readable annotations and database-matched scientific names for metabolomic features Related to STAR Methods

Document S2. Article plus supplemental information
