# Supplementary material for: Alterations of the host-lung microbiome metasystem in systemic inflammatory response syndrome is associated with secondary pneumonia
Source: Cell Rep Med. 2026 Jul 16;7(8):102919. doi: 10.1016/j.xcrm.2026.102919 (PMC13522790; doi:10.1016/j.xcrm.2026.102919)
Supplement: Supplementary file 1 [file mmc1.pdf]

Cell Reports Medicine, Volume *7*

Supplemental information

Alterations of the host-lung microbiome metasystem in systemic inflammatory response syndrome is associated with secondary pneumonia

Debajyoti Sinha, Melanie Petrier, Florian Pierre Martin, Cecile Poulain, Delphine Flattres Duchaussoy, Chiara Alberti, Stefanie Kreutmair, Jonas Schmid, Susanne Unger, Athanasios Ziogas, Despoina Koulenti, Laia Fernández-Barat, Antoni Torres, Cecile Braudeau, Regis Josien, Burkhard Becher, Mihai G. Netea, Robert P. Dickson, Emmanuel Montassier, Jeremie Poschmann, and Antoine Roquilly

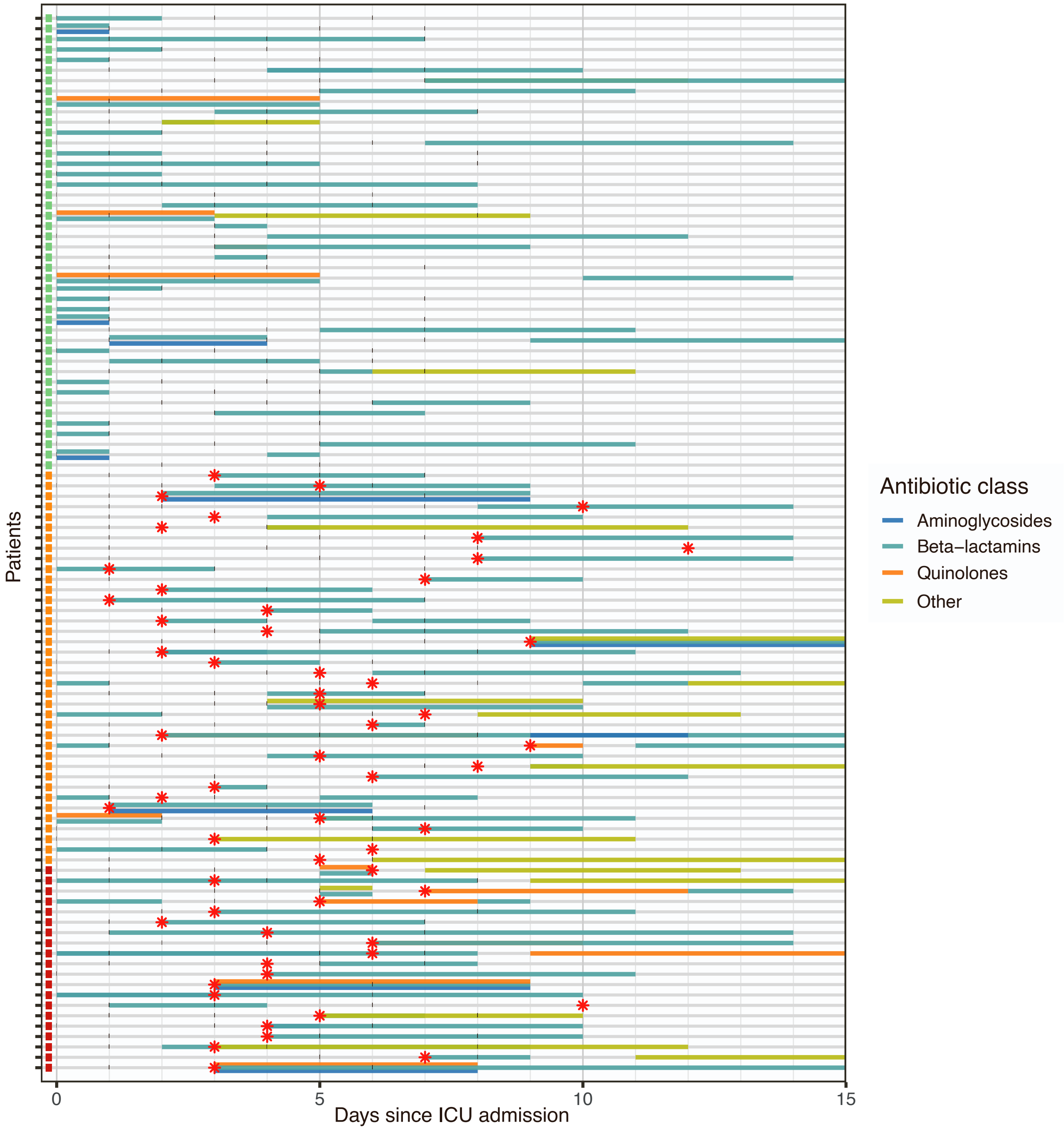

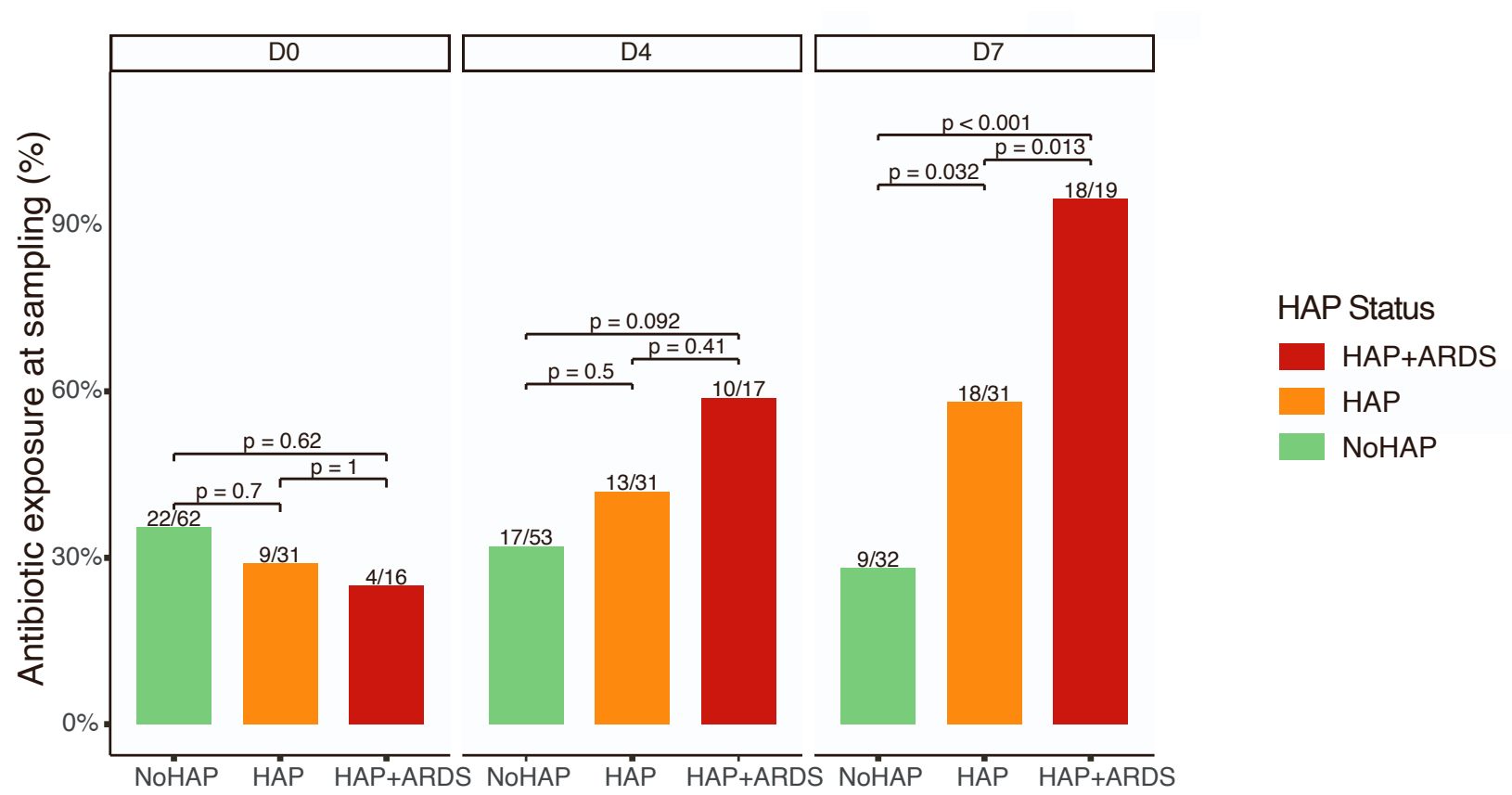

Figure S1. Antibiotic exposure during ICU admission of ptients from IBIS chohort (related to Figure 1). Overlapping courses within the same patient are vertically offset. Each row is a patient and left-side bars indicate HAP status. Vertical pipe (|) indicates the day of sample collection. The day of HAP diagnosis is indicated by *. Antibiotic exposure is shown from ICU admission to day 15. Antibiotic exposure to sampling timepoints and its association with clinical labels in IBIS cohort. Statistical significance was assessed Kruskal Wallis test.

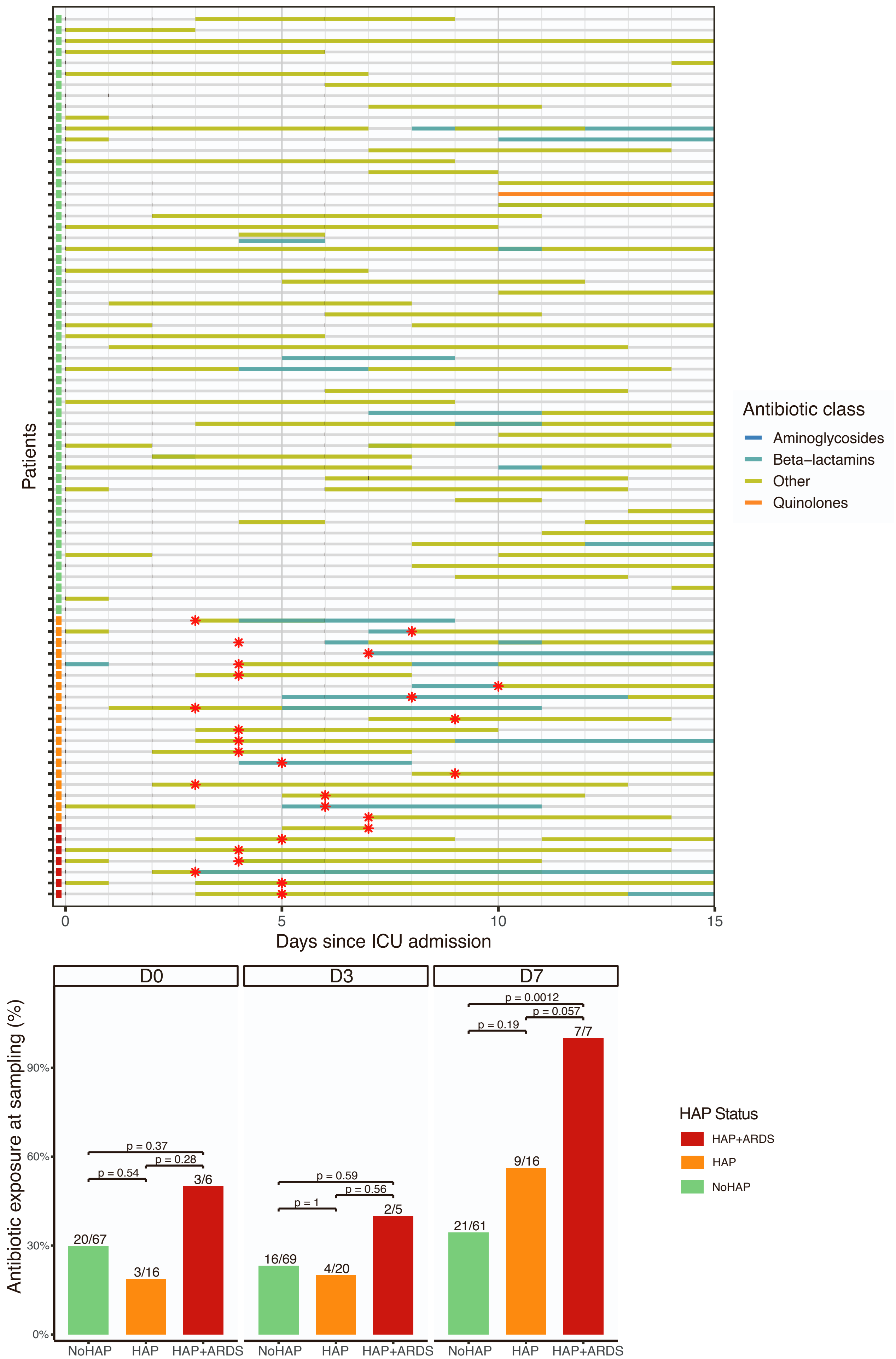

Figure S2. Antibiotic exposure during ICU admission of ptients from Prev-HAP chohort (related to Figure 1).

Overlapping courses within the same patient are vertically offset. Each row is a patient and left-side bars indicate HAP status. Vertical pipe (|) indicates the day of sample collection. The day of HAP diagnosis is indicated by *. Antibiotic exposure is shown from ICU admission to day 15. Antibiotic exposure to sampling timepoints and its association with clinical labels in Prev-HAP cohort.

Statistical significance was assessed Kruskal Wallis test.

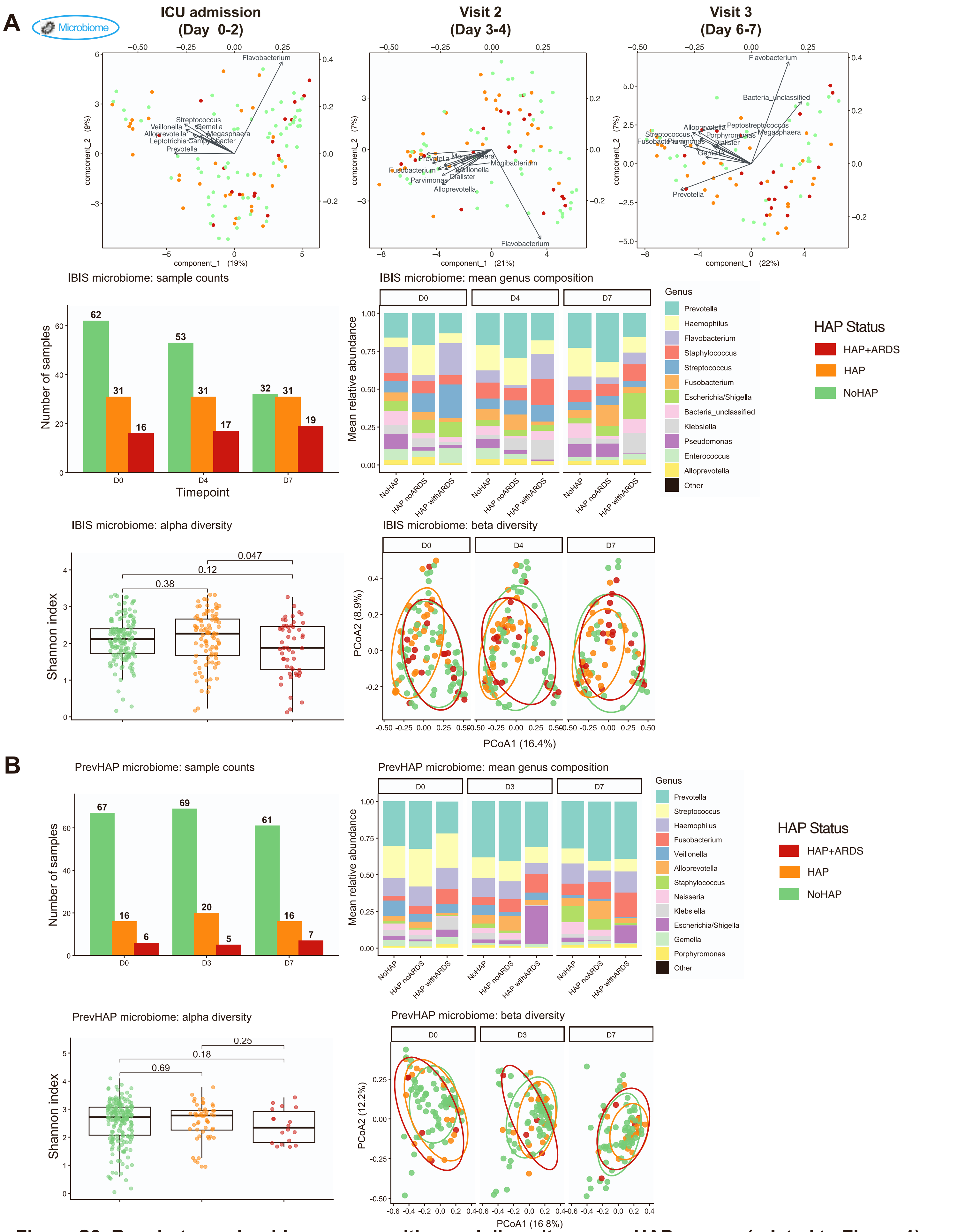

Figure S3. Respiratory microbiome composition and diversity across HAP groups (related to Figure 1).

A. IBIS cohort micriobiome data description: PCA biplot of samples coloured by HAP status and vector of genues level features at each timepoint in IBIS cohort; count of samples within each timepoint ; Top 12 genus level microbiome compositions by timepoint and HAP status; alpha and beta diversity. **B.** Micriobiome data description in the Prev-HAP cohort: Count of samples within each timepoint ; Top 12 genus level microbiome compositions by timepoint and HAP status; alpha and beta diversity.
Shanon index are presented as median value (25-75th percentile) and min-max values. The ellipses represent 95%confidence intervals.
Statistical significance was assessed Kruskal Wallis test.

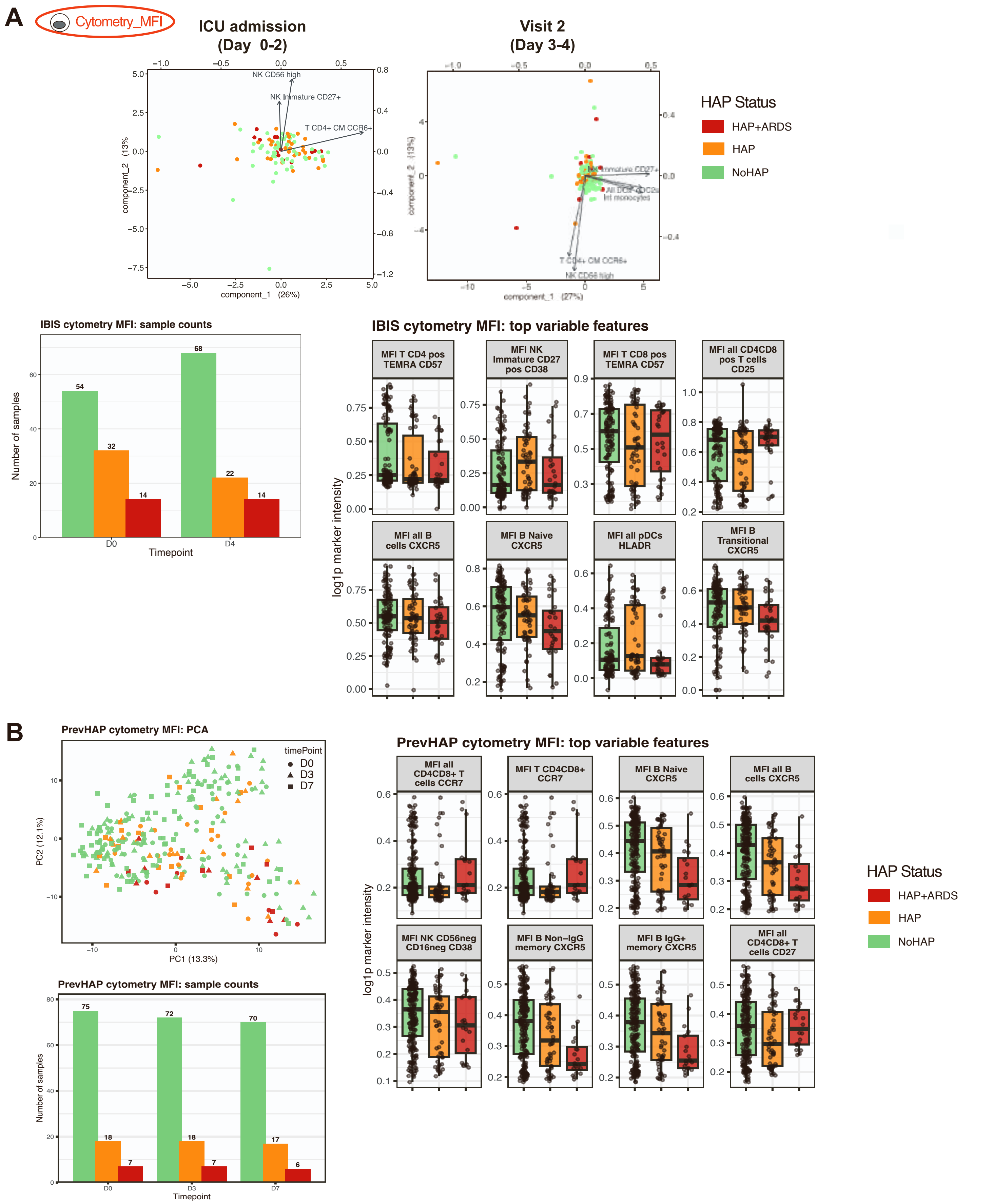

Figure S4. Blood immune-cell phenotype by cytometry MFI across HAP groups (related to Figure 1).

A. Descriptive data representations of spectral flow cytometry analysis (MFI) of PBMC in IBIS cohort. Samples were collected in critically ill patients at day 0 and day 3-4 after ICU admissions. PCA coordinates and biplot of samples at each timepoint in IBIS cohort; count of samples within each timepoint; intensity levels of top 8 ranked by variance cytometry MFI features, all coloured by HAP status. **B.** Descriptive data representations of spectral flow cytometry analysis (MFI) of PBMC in Prev-HAP cohort : PCA of all samples, count of samples within each timepoint; intensity levels of top 8 ranked by variance cytometry MFI features, coloured by HAP status

Data are represented median (25-75th percentile) and min-max values.

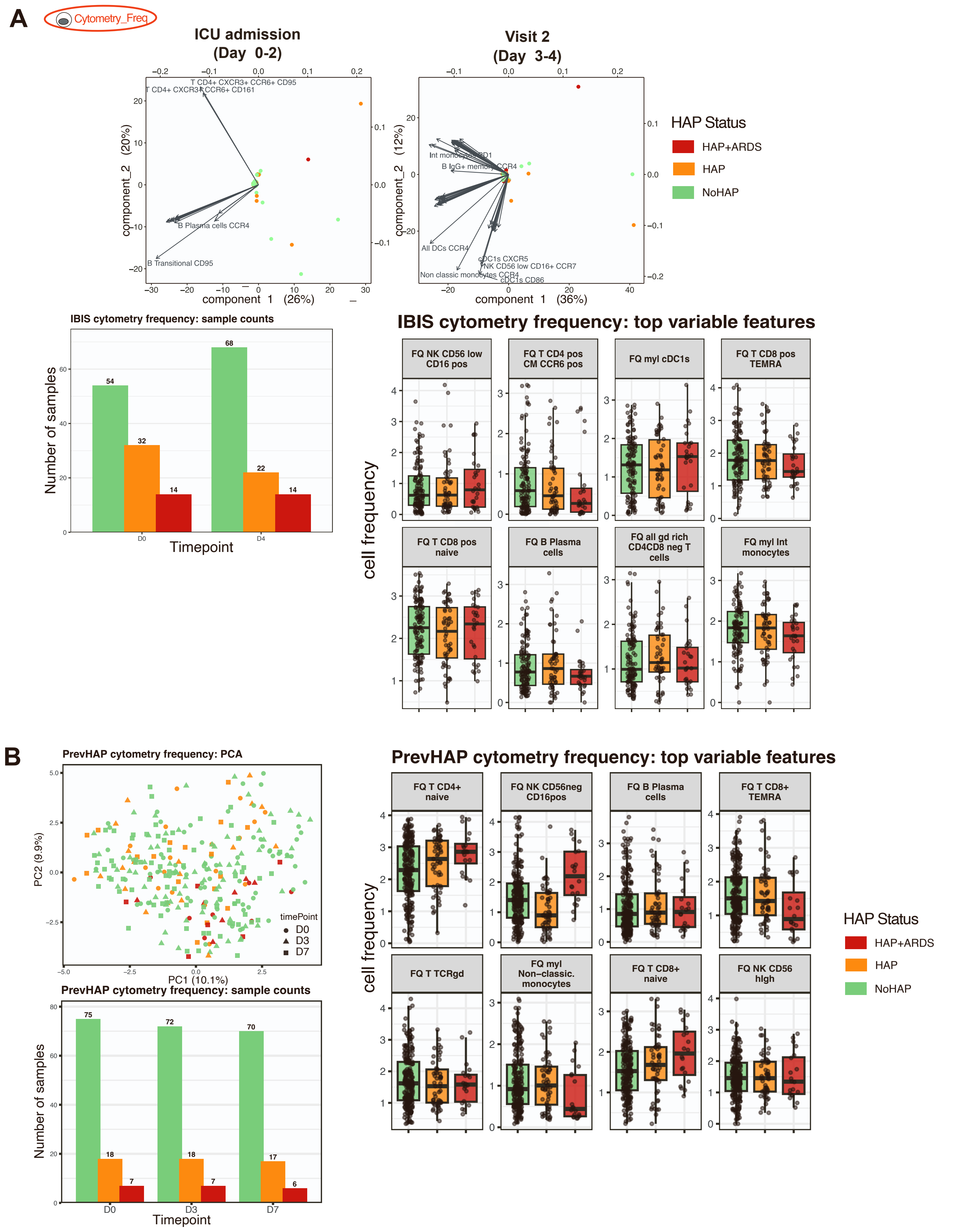

Figure S5. Blood immune-cell frequency profiles across HAP groups (related to Figure 1).

A. Descriptive data representations of spectral flow cytometry analysis: cell frequency (FQ) of PBMC in IBIS cohort. Samples were collected in critically ill patients at day 0 and day 3-4 after ICU admissions. PCA coordinates and biplot of samples at each timepoint in IBIS cohort; count of samples within each timepoint; cell frequency of top 8 ranked by variance cytometry FQ features, all coloured by HAP status. **B.** Descriptive data representations of spectral flow cytometry analysis (FQ) of PBMC in Prev-HAP cohort : PCA of all samples, count of samples within each timepoint; intensity levels of top 8 ranked by variance cytometry MFI features, coloured by HAP status.
Data are represented median (25-75th percentile) and min-max values.

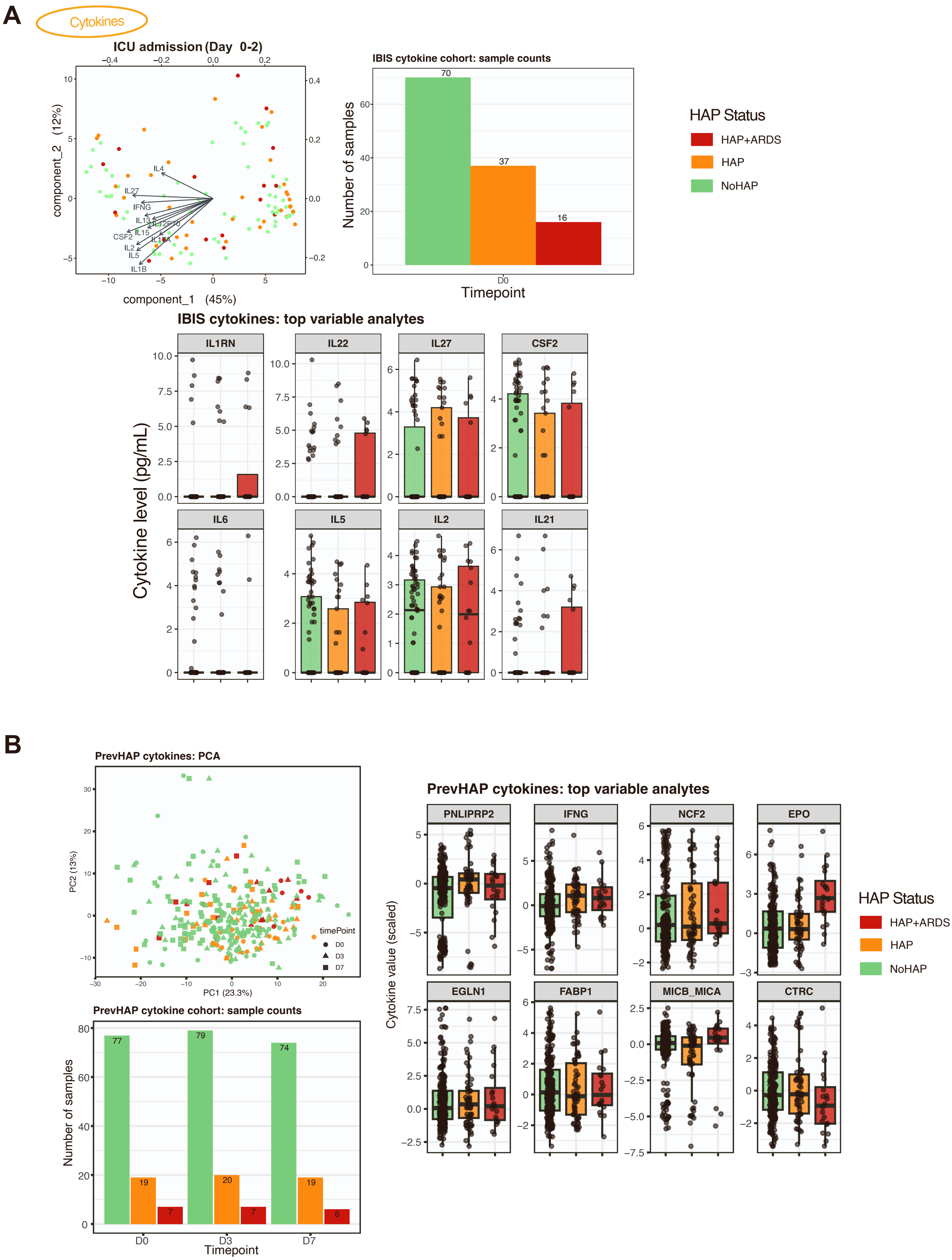

Figure S6. Circulating cytokine profiles across HAP groups related to Figure 1).

A. Descriptive data representations of cytokine blood levels measured by 34-plex Luminex assay in IBIS cohort. Samples were collected in critically ill patients at day of (D0) ICU admissions. PCA coordinates of and biplot of samples at D0; count of samples at D0; raw concentration levels of top 8 cytokines ranked by variance - all coloured by HAP status. **B.** Descriptive data representations of blood cytokines by Olink in Prev-HAP cohort : PCA of all samples, count of samples at D0; scaled intensity levels of top 8 cytokines ranked by variance, all coloured by HAP status. Data are represented median (25-75th percentile) and min-max values.

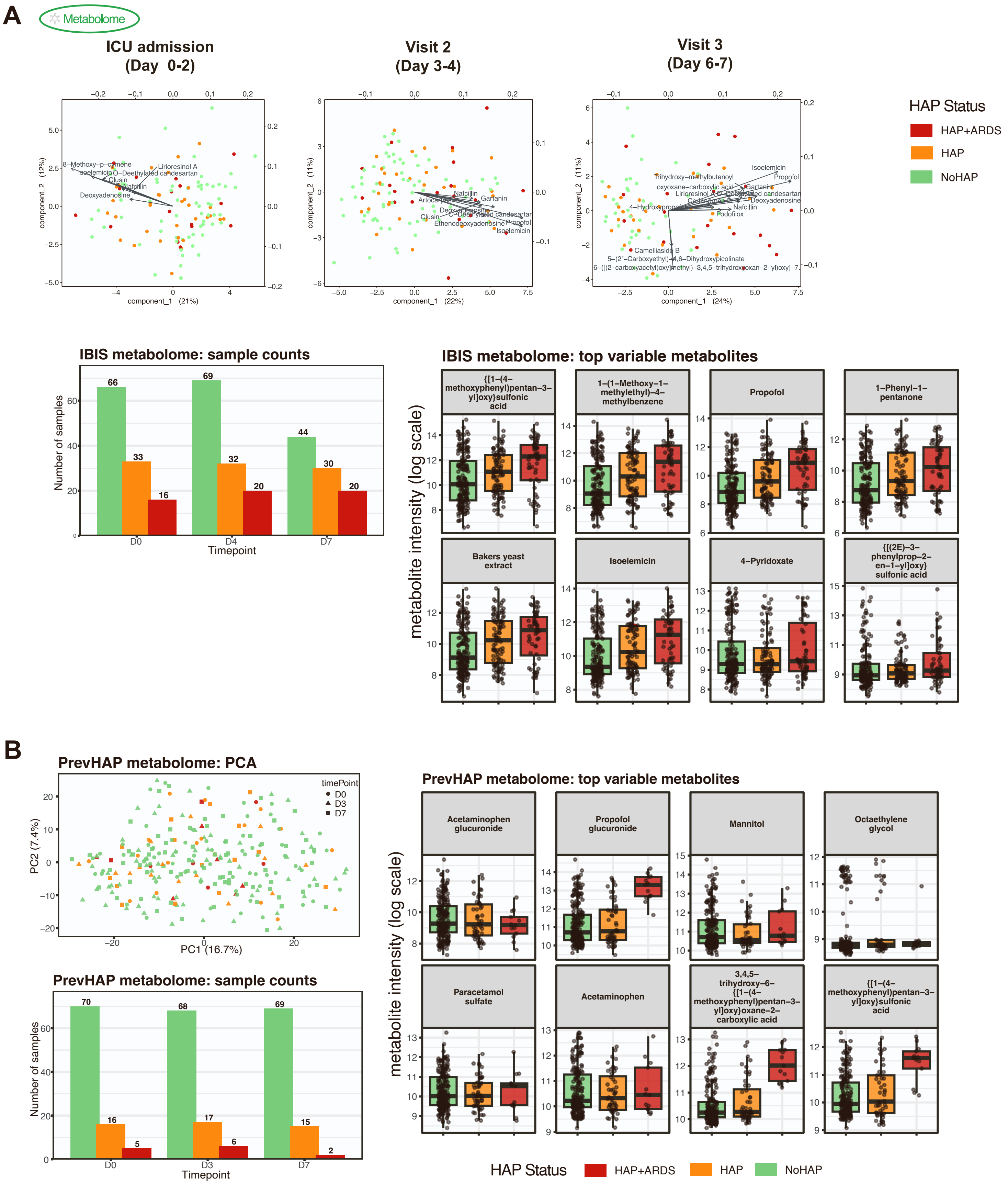

Figure S7. Plasma metabolome profiles across HAP groups (related to Figure 1).

A. Descriptive data representations of plasma metabolome in IBIS cohort. Samples were collected in critically ill patients at day 0-2, day3-4 and day 6-7 after ICU admissions. PCA coordinates of and biplot of samples for each timepoint; count of samples for each timepoint; Top 8 metabolites ranked by variance - all coloured by HAP status. **B.** Descriptive data representations of plasma metabolome in Prev-HAP cohort : PCA of all samples, count of samples within each timepoint; scaled intensity levels of top 8 metabolites ranked by variance, all coloured by HAP status. Data are represented median (25-75th percentile) and min-max values.

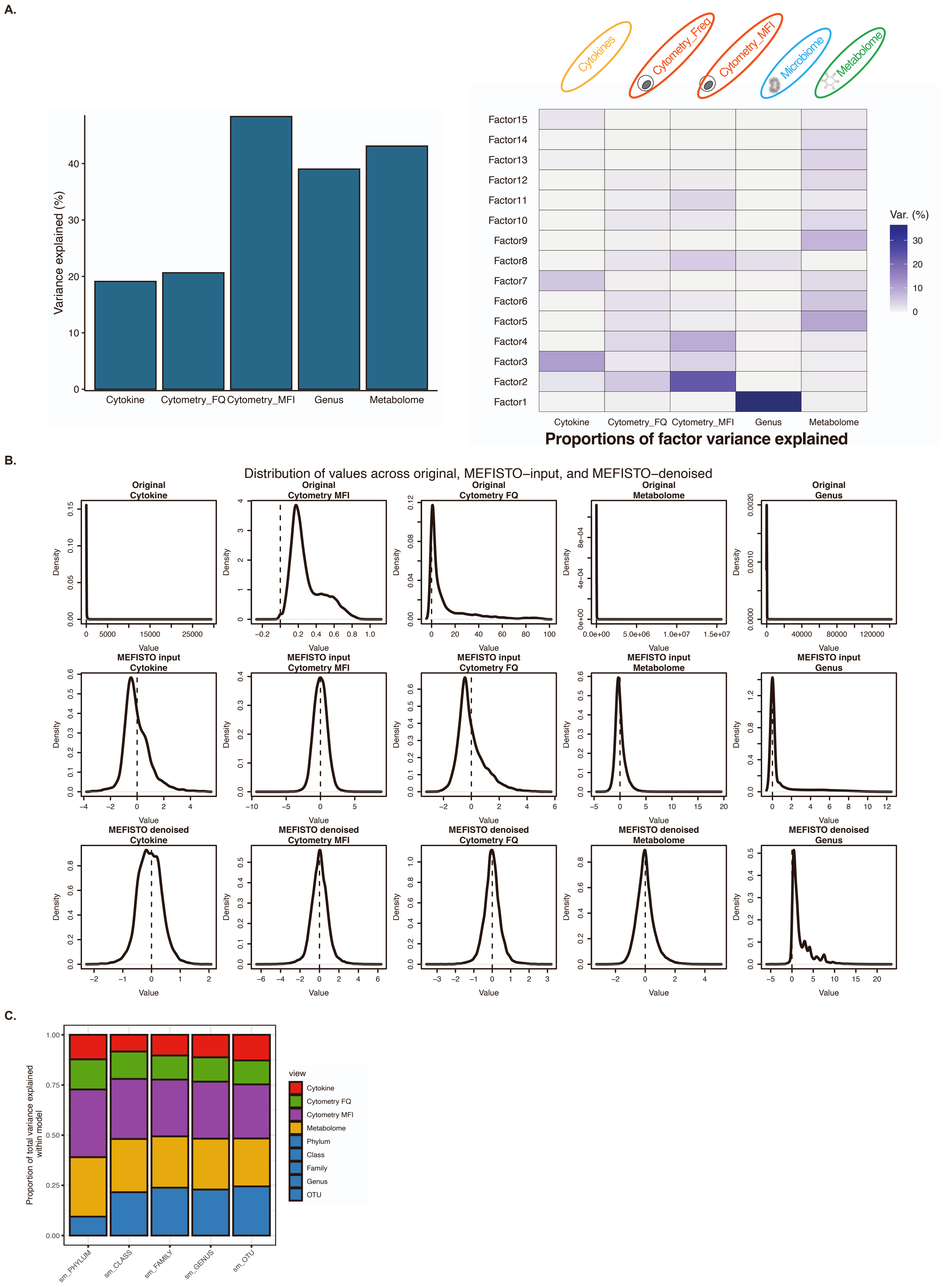

Figure S8. MEFISTO model structure, preprocessing, and variance across multi-omic layers (related to Figure 2).

A. List of 15 factors identified by MEFISTO pipeline. Proporition of factor variance explained by cytometry data (_MFI, and _Frequency), respiratory microbiome, blood cytokines and metabolome.
B. Density distribution of each omic data as original, preprocessed input for MEFISTO and final denoised model output ineach row respectively.
C. Omic variations captured in each model with different taxonomic levels representing the respiratory microbiome data.

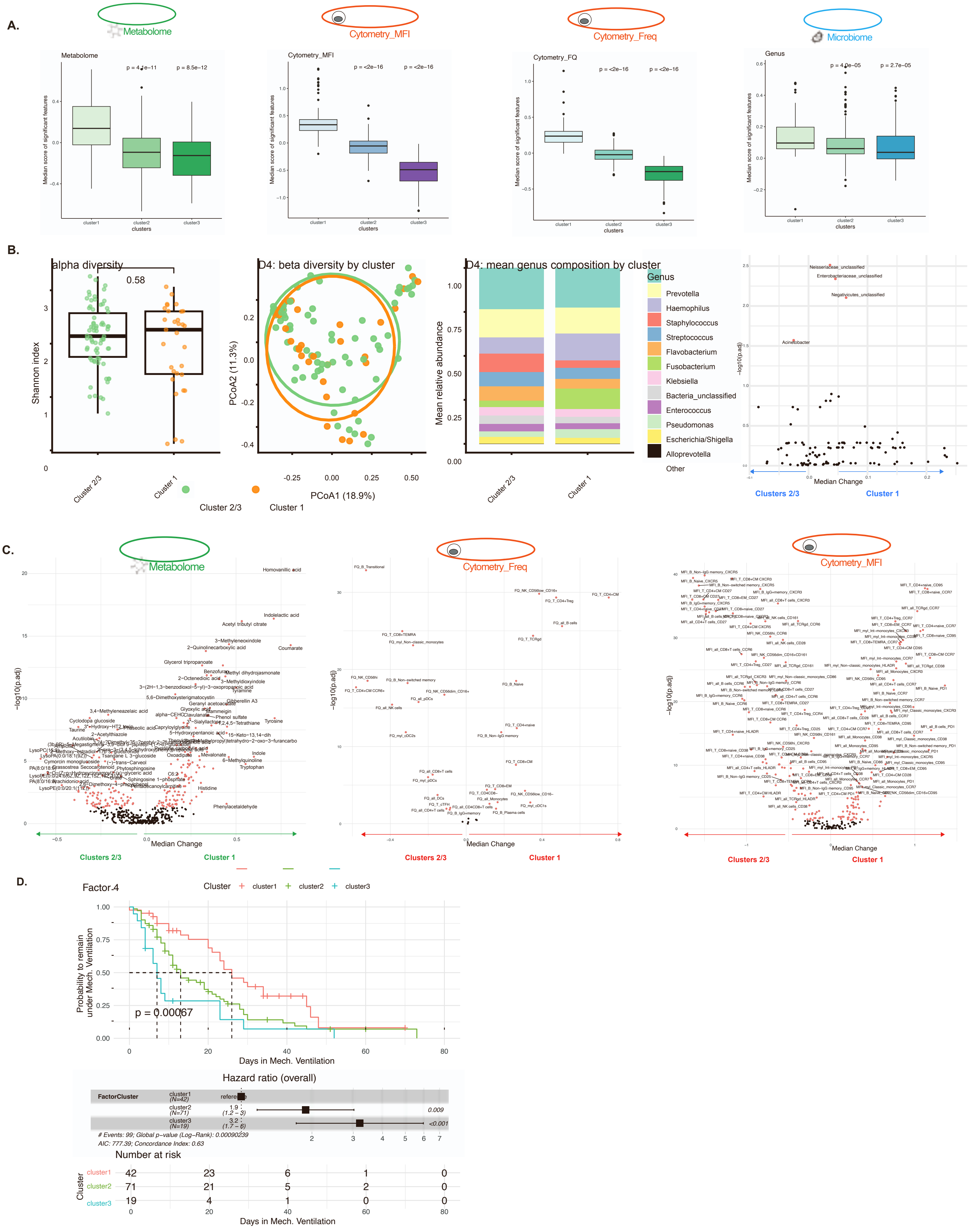

Figure S 9. Multi-omic features defining Factor 4 clusters and their association with survival (related to Figure 3).

A. Median scores of significant features of the microbiome, metabolome and flow cytomtry in Factor 4 clusters. Data are presented as median value (25-75th percentile) and min-max values.

B. Descriptive microbiome plots Cluster 1 vs Cluster 2/3. Shanon index are presented as median value (25-75th percentile) and min-max values. The ellipses represent 95%confidence interval

C. Volcano plots of metabolites, cytometry features (_Freq and _MFI), with different blood levels between cluster 1 versus clusters 2-3.

D. Kaplan Meier curves showing the probability of ICU hospitalisation in Factor 4 clusters.

Statistical significance was assessed (A) Kruskal Wallis test, (B) Wilcoxon rank sum test or (D) by the log rank for the survival curves

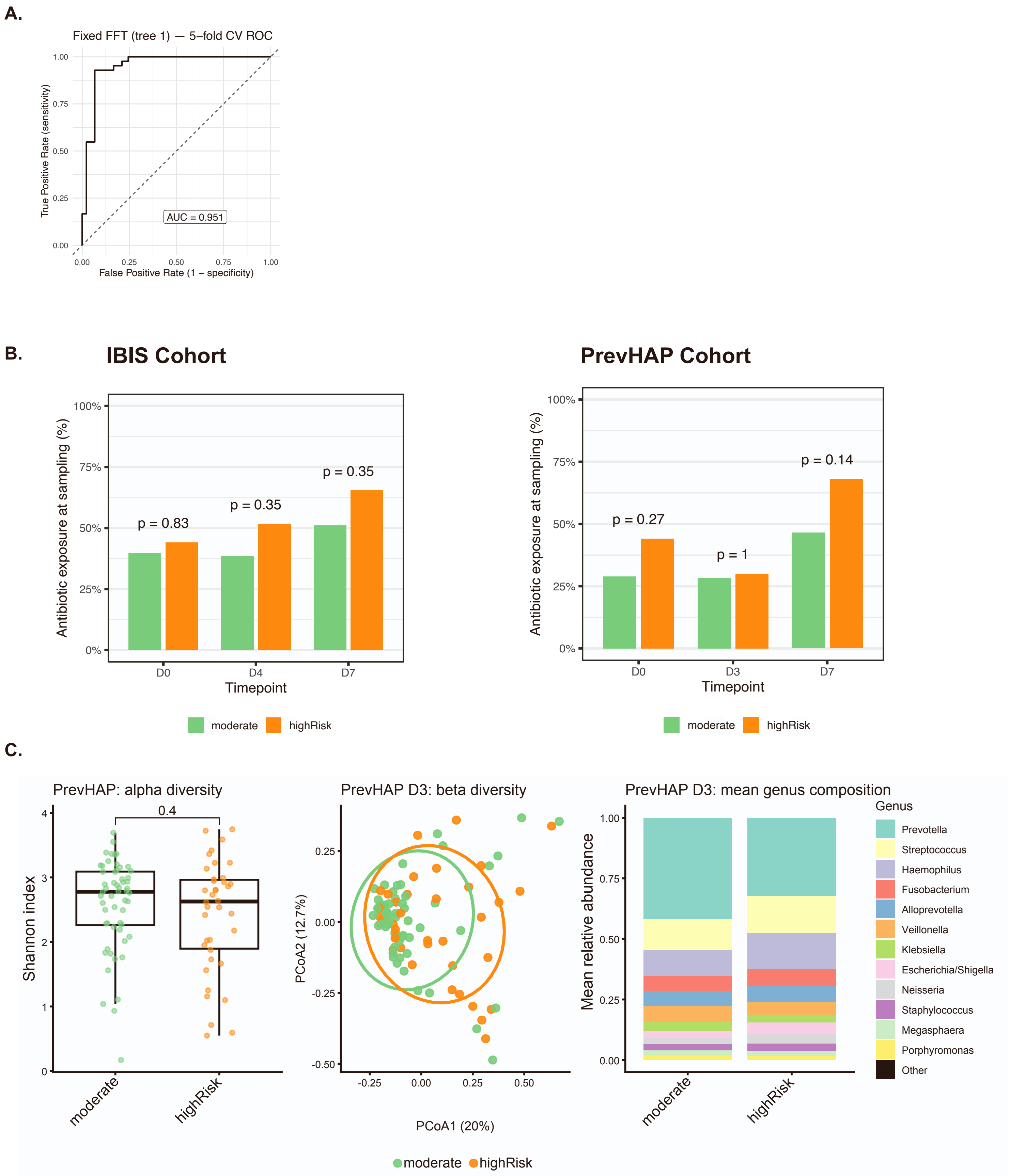

Figure S10. Validation of FFT high-risk classification and microbiome features in Prev-HAP (related to Figure 4).

A. ROC curve of the FFT-based score for identification of Factor 4 cluster in the IBIS cohort.
B. Percentage of samples exposed to an antibiotic on the day or immediately before sampling.
C. Genus level metrices in PrevHAP cohort by the predicted risk groups. Shanon index are presented as median value (25-75th percentile) and min-max values. The ellipses represent 95%confidence interval.
Statistical significance was assessed by Wilcoxon rank sum test.

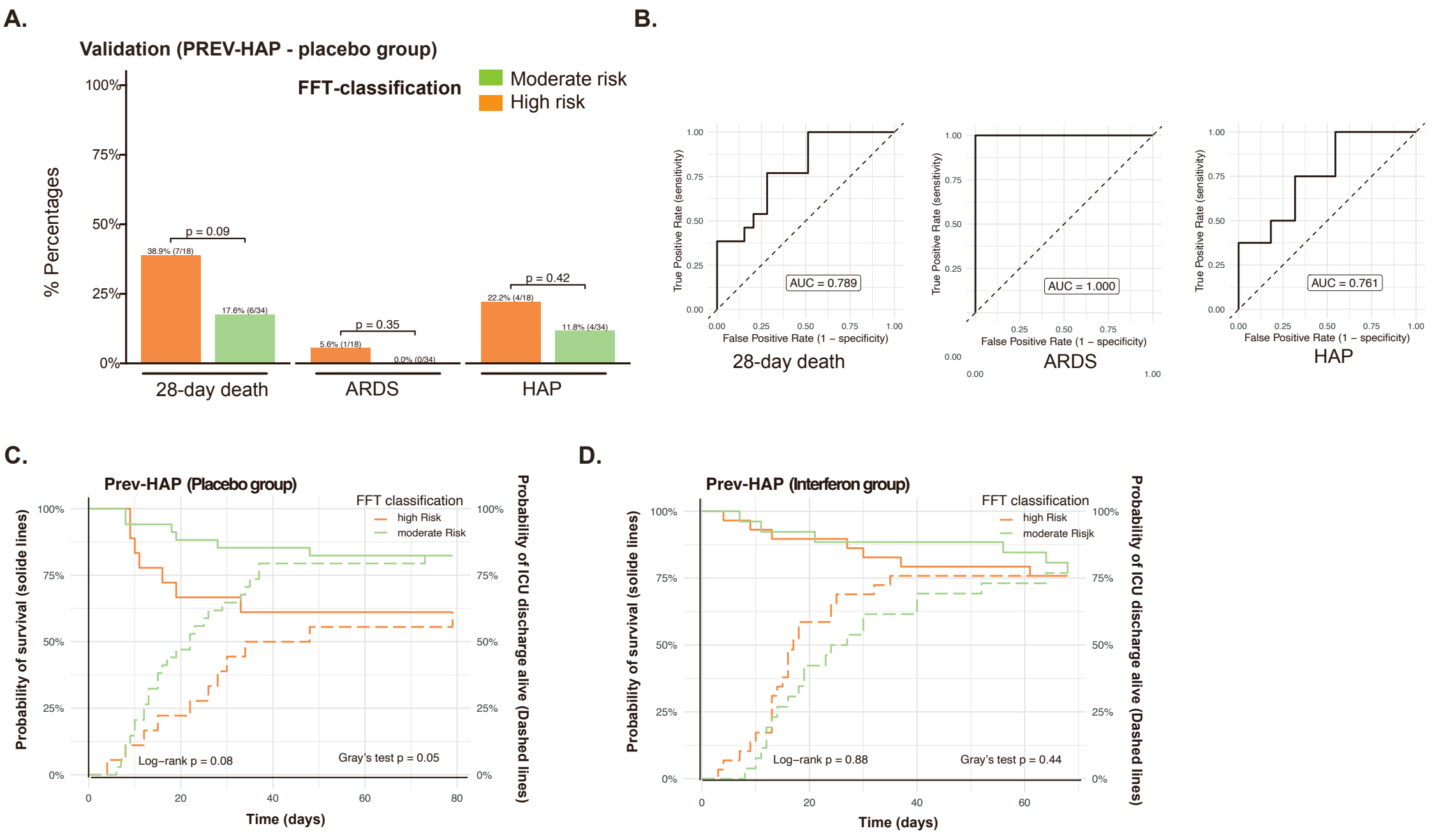

Figure S11. Clinical outcome validation of FFT risk groups in Prev-HAP. Related to Figure 3 (related to Figure 5).

(A) Frequencies of 28-day death, ARDS and HAP in moderate and high risk patients of the placebo group. (B) ROC curves of the FFT-based classification for 28-day death, ARDS and HAP in the placebo group. (C) Cumulative incidence curves for ICU discharge alive and survival curves in the placebo and (D) interferon-g groups stratified by FFT score for host-lung microbiome dysbiosis severity. Statistical significance was assessed (A) by Chi-squared Test (C-D) the log rank for the survival curves and Grey's test for the cumulative incidence function (discharged alive) curves.
